# Patient-Specific Computational Fluid Dynamics of Pulmonary Arterial Hemodynamics in Dogs with and Without Pulmonic Stenosis

**DOI:** 10.3390/ani16182842

**Published:** 2026-09-09

**Authors:** Carla Zamora-Perarnau, Mauro Malvè, Patricia Sebastián-Marcos, Rocío Fernández-Parra

**Affiliations:** 1 Doctoral School, Catholic University of Valencia San Vicente Mártir, San Agustín Square, 3, 46002 Valencia, Spain; 2 Department of Small Animal Medicine and Surgery, Faculty of Veterinary Medicine and Experimental Sciences, Catholic University of Valencia San Vicente Mártir, Quevedo Street 2, 46001 Valencia, Spain; patricia.sebastian@ucv.es (P.S.-M.); rocio.f.parra@ucv.es (R.F.-P.); 3 Veterinary Referral Hospital UCV, Catholic University of Valencia San Vicente Mártir, Pérez Galdós Street 51, 46018 Valencia, Spain; 4 Department of Engineering, Public University of Navarre (UPNA), Campus de Arrosadía s/n, 31006 Pamplona, Spain; mauro.malve@unavarra.es; 5 Biomedical Research Networking Center in Bioengineering, Biomaterials, and Nanomedicine (CIBER-BBN), Monforte de Lemos, 3-5, 28029 Madrid, Spain; 6 Institute for Advanced Materials and Mathematics (INAMAT^2^), Public University of Navarre (UPNA), Edificio Jerónimo de Ayanz, Campus de Arrosadía s/n, 31006 Pamplona, Spain

**Keywords:** computational fluid dynamics, impedance-based pressure waveforms, pulmonary artery hemodynamics, pulmonic stenosis, vascular resistance, wall shear stress indicators

## Abstract

Pulmonic stenosis (PS) is one of the most common congenital cardiac diseases in dogs. Although echocardiography and computed tomographic angiography (CTA) provide valuable diagnostic and anatomical information, the hemodynamic consequences of PS remain incompletely characterized. This study describes patient-specific pulmonary artery hemodynamics in dogs with and without PS using computational fluid dynamics (CFD). Contrast-enhanced CTA and echocardiographic data from five dogs with PS and five control dogs without PS were used to reconstruct three-dimensional pulmonary artery geometries and generate patient-specific CFD models. Pressure drop (Δp), blood flow velocity, mean wall shear stress (WSS), time-averaged WSS (TAWSS), oscillatory shear index (OSI), relative residence time (RRT), and pulmonary artery resistance were quantified and compared between groups. Dogs with PS exhibited markedly increased transvalvular pressure gradients, peak flow velocities, and pulmonary artery resistance, together with high-velocity jets and localized post-stenotic recirculation that were absent in control dogs. Mean WSS and TAWSS tended to be higher in the PS group, whereas OSI values were similar between groups. In contrast, RRT was significantly lower (*p* = 0.032) in dogs with PS. These findings demonstrate that patient-specific CFD enables detailed characterization of pulmonary artery hemodynamics in canine PS, providing information that complements conventional imaging. Although its clinical utility in veterinary medicine has yet to be fully established, CFD can provide valuable insight into flow behavior and disease pathophysiology; however, its ability to yield precise, clinically applicable quantitative values remains limited. This approach shows promise for future applications in the clinical evaluation of PS and other cardiovascular diseases, although further studies are warranted to determine its clinical relevance in veterinary medicine.

## Simple Summary

Pulmonic stenosis is a congenital heart disease caused by the narrowing of the pulmonary valve, leading to a flow obstruction between the right ventricle and main pulmonary artery. Although echocardiography and computed tomography are routinely used to diagnose this condition, they do not provide complete information about how blood moves through the affected vessels. In this study, we created three-dimensional computer models of the pulmonary arteries from medical images of dogs with and without pulmonic stenosis and simulated blood flow under realistic conditions. Dogs with pulmonary stenosis exhibited markedly higher blood flow velocities, greater pressure drops across the stenotic valve, and more disturbed flow patterns compared with dogs with a normal pulmonary valve. In particular, areas of swirling blood flow were observed beyond the narrowing, which altered the forces acting on the vessel walls. These findings improve our understanding of the changes in blood flow caused by pulmonic stenosis and demonstrate that computer-based blood flow simulations can provide valuable information that complements conventional imaging techniques. This approach may contribute to the future assessment of cardiovascular diseases in veterinary medicine and help improve treatment planning for dogs with congenital heart disease.

## 1. Introduction

Pulmonic stenosis (PS) is one of the most common congenital cardiac diseases in dogs [1]. According to the location of the obstruction, PS can be classified as subvalvular, valvular, or supravalvular. Valvular stenosis is the most common form and has been further classified into two anatomical subtypes: type A and type B. In type A, the pulmonary annulus has a normal diameter, whereas the valve leaflets show varying degrees of thickening and fusion, resulting in systolic doming of the valve, an eccentric valvular opening, and variable reduction of the effective cross-sectional area. Post-stenotic dilatation of the main pulmonary artery (MPA) is commonly observed in these cases. In contrast, type B is characterized by a hypoplastic pulmonary annulus, thickened and relatively immobile valve leaflets, and post-stenotic dilatation is uncommon [2]. It is most frequently observed in brachycephalic breeds, although many dogs display a combination of morphological features consistent with both stenotic types [3].

Diagnosis is primarily based on echocardiography. The most common echocardiographic method for determining PS severity—and the only one described in veterinary guidelines [2]—is the Doppler-derived maximum velocity of blood flow across the stenotic PV. Maximum velocity is converted to the PV maximum pressure gradient using the simplified Bernoulli equation (pressure gradient = 4 V^2^) [4]. According to current recommendations, PS is classified as mild when the peak pressure gradient is less than 50 mmHg, moderate when it ranges between 50 and 80 mmHg, and severe when it exceeds 80 mmHg [2]. The advantages of this method include its ease of acquisition and independence from body size. However, in addition to the degree of stenosis, pressure gradients are largely influenced by transvalvular flow [4]. Other described methods include the velocity time integral ratio and the velocity ratio, as well as pulmonary valve (PV) area indexed to body surface area, which is theoretically less dependent on transvalvular flow [4]. Reduced cardiac output caused by, for example, sedatives, anesthetics, negative inotropic drugs, or ventricular systolic dysfunction may underestimate stenosis severity. Conversely, high cardiac output states such as stress/anxiety, anemia, heightened sympathetic tone, or after interventional states might overestimate stenosis severity [5]. An integrative approach to assessing PS severity should combine pressure gradient-based methods with at least one less flow-dependent measure to provide additional clinically relevant information and help guide treatment decisions, particularly in dogs with right ventricular systolic dysfunction [4].

Certain breeds, particularly Boxers and English Bulldogs, are predisposed to PS [3,6]. In addition, English Bulldogs have an increased prevalence of congenital coronary artery anomalies, most commonly a single right coronary ostium with an anomalous prepulmonic left coronary artery (type R2A anomaly). Identification of coronary anatomy before pulmonary balloon valvuloplasty (PBV) is therefore essential, as these anomalies may contraindicate the procedure or require modification of the therapeutic approach [6]. Pulmonary balloon valvuloplasty is currently considered the treatment of choice for dogs with moderate to severe PS [7,8]. Consequently, detailed preoperative assessment of the PV and coronary anatomy is of vital importance.

Aortic root angiography has been recommended prior to PBV to identify coronary artery anatomy and assess for the presence of a coronary anomaly. However, this procedure requires arterial access and is often performed via a cut-down procedure of the carotid or femoral artery [7]. Electrocardiographic- (ECG) and non-ECG-gated computed tomographic angiography (CTA) has emerged as a rapid and minimally invasive alternative to conventional angiography for the evaluation of coronary artery anatomy and the detection of associated vascular anomalies before intervention [6,9,10]. An ECG-gated technique links image acquisition to a specific phase of the cardiac cycle [11]. However, ECG-gating is not widely available in veterinary practice [12]. A study published in 2015 compared standard morphological echocardiographic measurements with measurements obtained using non-ECG-gated contrast-enhanced cardiac CT in dogs. The study concluded that no equivalence was found between the remaining echocardiographic and CT measurements. Therefore, non-ECG-gated CT has limitations for the quantitative assessment of cardiac size, partly because of poor visualization of internal cardiac diameter boundaries, resulting from cardiac motion artifacts and the use of 4-slice CT [12].

Although echocardiography and advanced imaging techniques provide valuable anatomical and functional information, they offer limited insight into the complex hemodynamic alterations generated by PS. Computational fluid dynamics (CFD) is an in silico technique widely used in human cardiovascular research that enables numerical quantification and visualization of hemodynamic variables—such as pressure, velocity, wall shear stress (WSS)—many of which cannot be measured directly in vivo [13]. Furthermore, it allows the evaluation of clinically relevant parameters, including systemic and end-organ oxygen delivery (particularly cerebral and myocardial perfusion) while reducing the need for invasive procedures [14]. Since these biomechanical forces play a fundamental role in the initiation and progression of numerous cardiovascular diseases, CFD has become an essential tool for clinical investigation. By developing patient-specific computational models, researchers can accurately simulate complex blood flow behaviors and their direct interaction with the vessel wall [15].

Advances in medical imaging, image processing, and computational power have substantially increased the accessibility and clinical applicability of CFD over the last decade. Patient-specific simulations have contributed to the understanding of the hemodynamics associated with coronary artery disease, carotid and aortic atherosclerosis, congenital heart disease (including primary pulmonary vein stenosis), ventricular function, prosthetic and native heart valves, coronary stents, bypass grafting, and cardiovascular treatment planning [16,17,18]. More specifically, pulmonary artery stenosis has been investigated in the context of congenital heart disease, with particular emphasis on its hemodynamic impact. Computational fluid dynamics enables detailed, patient-specific characterization of the hemodynamic alterations associated with this condition, although computational time remains one of its main reported limitations [19].

In veterinary medicine, CFD has been increasingly applied to respiratory diseases. Previous studies have investigated airflow dynamics in dogs with brachycephalic obstructive airway syndrome [20,21,22]; compared airflow structures, pressure distributions, and airway resistance among canine breeds [23]; and modeled the transport, distribution, and deposition of inhaled pharmacological particles in the feline respiratory tract under different breathing conditions [24], using different devices [25], and in both healthy and asthmatic cats [26]. This technique has also been used to evaluate upper airway velocity, pressure, and resistance during general anesthesia in cats fitted with different airway devices [27]. However, to the authors’ knowledge, CFD has not previously been applied to investigate cardiovascular pathologies in clinical veterinary medicine.

The aim of this study was to develop patient-specific CFD models of dogs with and without PS by combining CTA and echocardiographic data to characterize pulmonary artery hemodynamics. We hypothesized that dogs with PS would exhibit significant alterations in blood flow patterns and hemodynamic parameters, including pressure drop (Δp), velocity, mean WSS, and WSS-derived indices, compared with dogs without PS. Furthermore, this study aimed to contribute to future research exploring the potential role of CFD in the investigation of cardiovascular diseases in clinical veterinary medicine, including its possible applications in treatment evaluation and surgical planning.

## 2. Materials and Methods

### 2.1. Animals and Ethical Approval

In this retrospective, descriptive, and comparative study, thoracic computed tomography (CT) scans of dogs performed as part of a diagnostic protocol at the Veterinary Referral Hospital UCV (Catholic University of Valencia San Vicente Mártir) between 2021 and 2025 were selected for analysis. All patients were drawn from the hospital’s clinical caseload. Computed tomography scans were performed as part of the diagnostic protocol in dogs without PS and, in dogs with PS, to evaluate the presence of aberrant coronary arteries before PBV. All dogs also underwent a comprehensive echocardiographic examination; in dogs without PS, echocardiography confirmed the absence of PV abnormalities, cardiac remodeling, evidence of pulmonary hypertension, and abnormalities in systolic or diastolic function. Dogs were classified into the non-pulmonic stenosis (N-PS) group if echocardiographic findings were not consistent with PS, whereas dogs with an echocardiographic diagnosis of PS were assigned to the PS group. Clinical signs and final diagnoses were also recorded, including the type of PS and any concomitant cardiac abnormalities in dogs with PS, or the final diagnosis in N-PS dogs. Exclusion criteria included marked motion artifacts, nondiagnostic CTA, other structural cardiac abnormalities, systemic diseases that could cause cardiac hemodynamic disturbances (such as severe anemia, hypoproteinemia, or systemic inflammatory conditions), and arrhythmias. All procedures were performed as part of standard veterinary clinical practice with owner consent, and the study was approved by the institutional ethics committee (CEEAUCV2101).

### 2.2. Image Acquisition and Analysis

All CT scans were performed using a multidetector 16-slice CT scanner (Siemens Somatom Scope, Munich, Germany) in helical scan mode. All dogs were positioned in sternal recumbency, and scans of the thorax were performed, from the level of C7 to the most cranial part of the liver. Computed tomography acquisition protocols and scan settings included a 512 × 512 matrix, a pitch of 0.95, a slice thickness of 1–3 mm, 120–150 mAs, 130 kV, and a patient-size-adjusted display field of view. Patients underwent CT acquisition during spontaneous breathing or mechanical ventilation, without breath-hold maneuvers. At the end of the CT examination, the dogs were supervised until they recovered or remained anesthetized for additional procedures, like balloon pulmonary valvuloplasty or other diagnostic investigations.

Anesthetic protocols were individualized for each patient and adjusted according to clinical requirements. In the N-PS group, premedication consisted of intravenous administration of butorphanol (Butomidor^®^, 10 mg/mL, VetViva Richter GmbH, Wels, Austria) at 0.3–0.4 mg/kg, methadone (Metasedin^®^, 10 mg/mL, Esteve Pharmaceuticals S.A., Valencia, Spain) at 1–2 mg/kg, or dexmedetomidine (Dexdomitor^®^, 0.5 mg/mL, Zoetis Spain S.L., Madrid, Spain) at 0.5–2.5 μg/kg. Anesthesia was induced with either propofol (Propofol Lipoven Fresenius^®^, 10 mg/mL, Fresenius Kabi Deutschland GmbH, Bad Homburg, Germany) at 2–4 mg/kg or alfaxalone (Alfaxan^®^ Multidose, 10 mg/mL, Zoetis Spain S.L., Madrid, Spain) at 1 mg/kg, in combination with either midazolam (Midazolam Normon EFG^®^, 5 mg/mL, Laboratorios Normon S.A., Madrid, Spain) at 0.2–0.3 mg/kg or ketamine (Ketabel^®^, 100 mg/mL, Fatro Ibérica, Sant Just Desvern, Spain) at 1 mg/kg.

In the PS group, all patients were premedicated with butorphanol at 0.3–0.4 mg/kg. Anesthesia was induced with either propofol at 3 mg/kg or alfaxalone at 1–2 mg/kg to effect, in combination with midazolam at 0.2–0.3 mg/kg. Anesthesia was maintained in all patients with sevoflurane (Sevotek^®^, 1000 mg/g, Laboratorios Karizoo, Caldes de Montbui, Spain) delivered in an oxygen/medical air mixture (FiO_2_ 50–60%), with the vaporizer set at 2.0–2.3%. Lactated Ringer’s solution (Lactato-Ringervet^®^, B. Braun VetCare, Melsungen, Germany) was administered at 2–3 mL/kg/h during the procedure and adjusted as needed. A dedicated anesthesiologist monitored anesthesia throughout the procedure until full recovery.

In cases of hypotension (mean arterial pressure [MAP] < 60 mmHg), the inhalational anesthetic concentration was adjusted and/or ephedrine (Hidrocloruro de Efedrina Kabi^®^, 30 mg/mL, Fresenius Kabi Deutschland GmbH, Bad Homburg, Germany) was administered at 0.05 mg/kg until normotensive values were restored.

Images were viewed using two DICOM (Digital Imaging and Communications in Medicine) viewers (Osirix MD v14.2.0 and Horos v4.0.1) with a soft tissue (window width [WW] = 120 HU (Hounsfield units) and window level [WL] = 40 HU) reconstruction algorithm.

**Figure 1:**
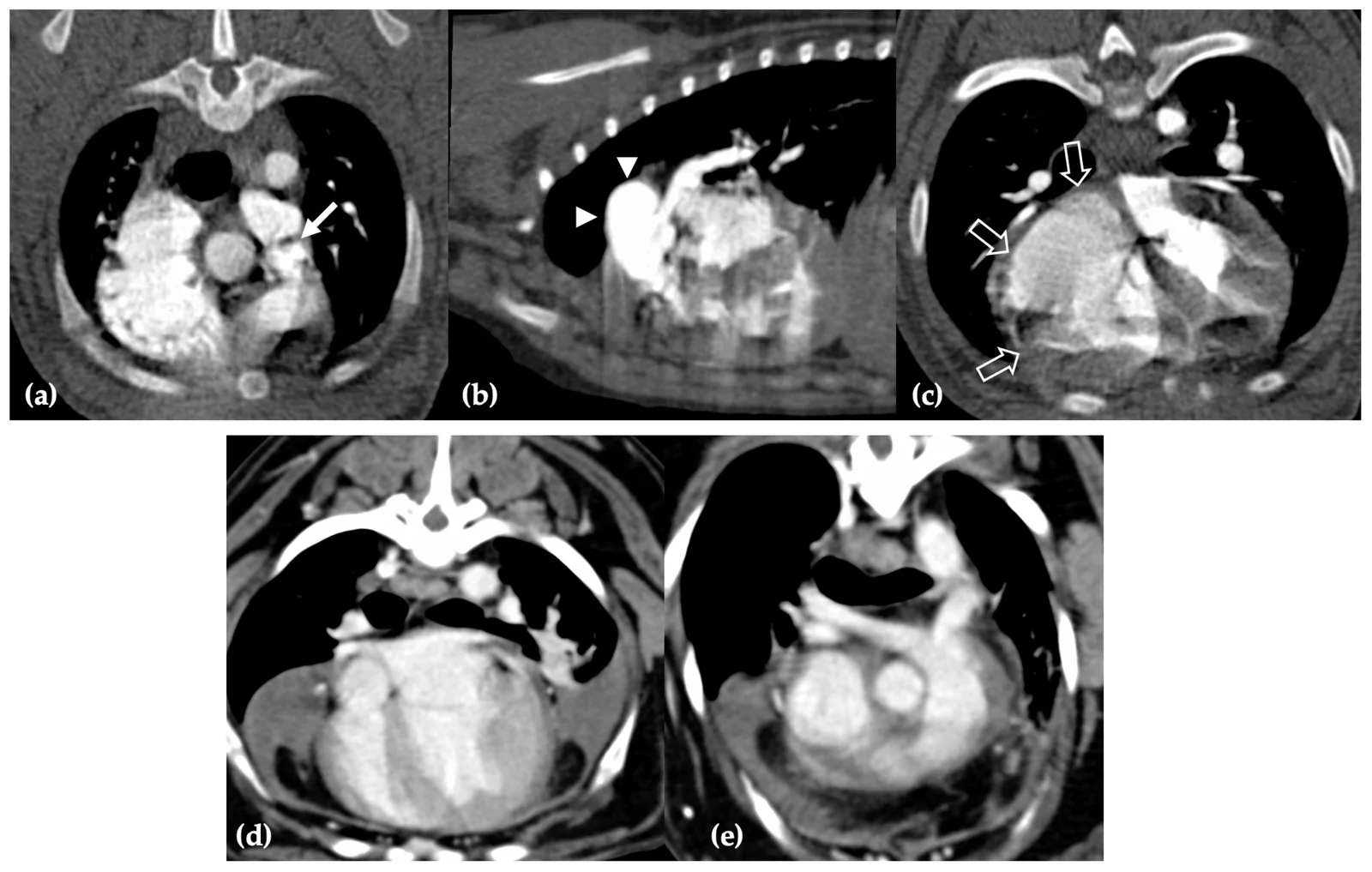
Computed tomography images in transverse (**a**,**c**–**e**) and sagittal (**b**) planes using a soft-tissue visualization algorithm; (**a**,**c**) from case #2 PS group, (**b**) from case #1 PS group, and (**d**,**e**) from case #3 N-PS group. Pulmonary valve stenosis (white arrow), post-stenotic dilation (white arrowheads), and dilation of the right cardiac chambers (open arrows) are observed. Normal cardiac silhouette and pulmonary trunk (**d**,**e**). PS: pulmonic stenosis; N-PS: non-pulmonic stenosis.

The appearance of the cardiac silhouette, cardiac chambers, and blood vessels was assessed on CT images. In animals with PS, the presence of PS (Figure 1a), poststenotic dilatation (Figure 1b), enlargement of the cardiac chambers (Figure 1c), and dilation of the pulmonary arteries were assessed. Dogs in the N-PS group had normal cardiac size, and no abnormalities of the blood vessels were identified (Figure 1d,e).

### 2.3. Geometrical Reconstruction and Numerical Discretization

**Figure 2:**
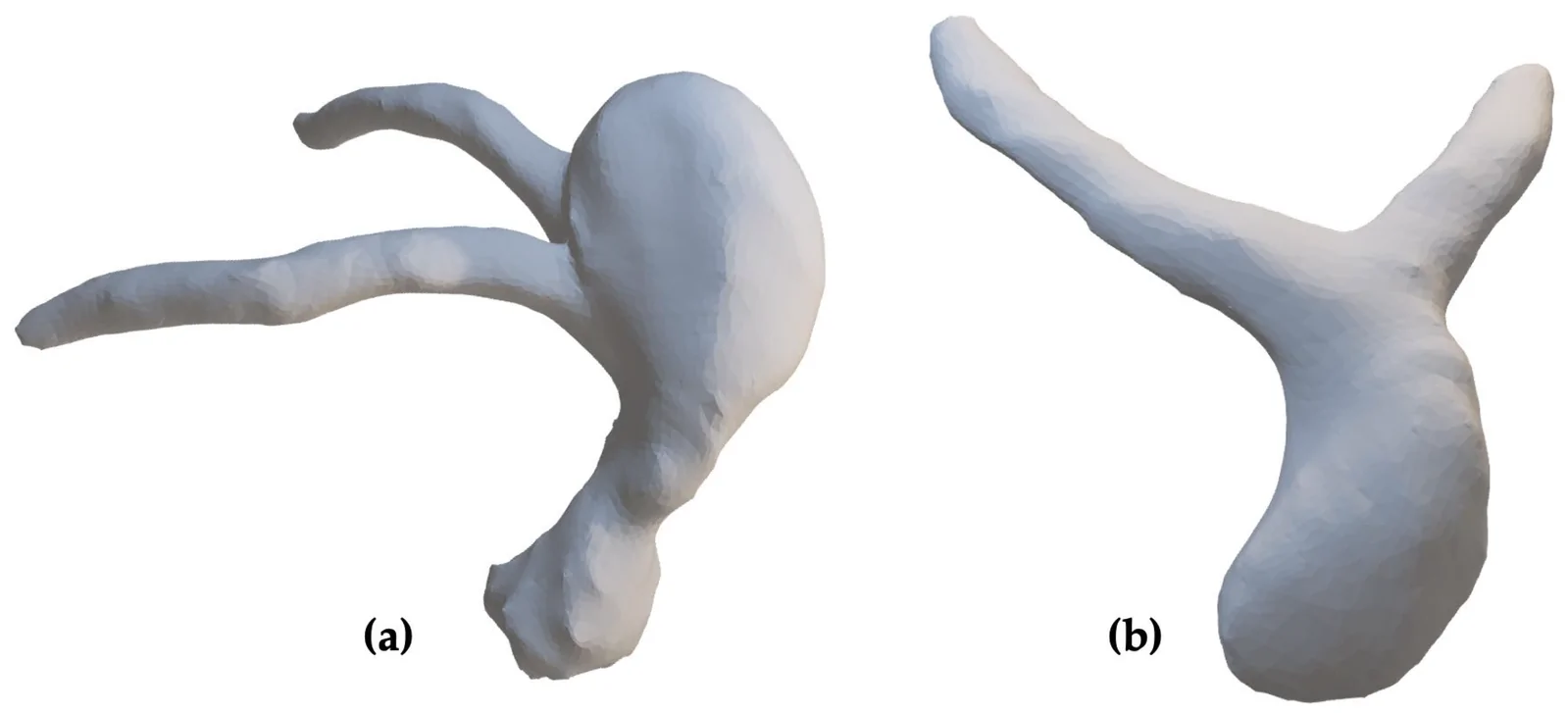
Patient-specific anatomical reconstructions generated from CT images using the image-based reconstruction software MIMICS. (**a**) Reconstruction of the RVOT, MPA, post-stenotic dilation, LPA and RPA in a dog with PS (PS; Case #1, PS group). (**b**) Reconstruction of the RVOT, MPA, LPA and RPA in a dog without PS (N-PS; Case #3, N-PS group). RVOT: right ventricular outflow tract; MPA: main pulmonary artery; LPA: left pulmonary artery; RPA: right pulmonary artery; PS: pulmonic stenosis; N-PS: non-pulmonic stenosis.

The DICOM datasets obtained from the CT scans were imported into the image-based reconstruction software MIMICS Core 28.0 (Materialise NV, Leuven, Belgium). Patient-specific geometries of the right ventricular outflow tract (RVOT), MPA, left pulmonary artery (LPA), and right pulmonary artery (RPA) were manually segmented and reconstructed for each dog. Representative reconstructed geometries of a dog with PS and a dog without PS are shown in Figure 2a and Figure 2b, respectively. The resulting geometries were exported as stereolithography (STL) files.

The STL models were subsequently imported into Rhinoceros 7 (Robert McNeel & Associates, Seattle, WA, USA) for geometry processing. Inlet and outlet sections were defined, and the pulmonary artery bifurcation was separated into the LPA and RPA. The processed surface geometries were then exported as STL files and imported into ANSYS ICEM CFD (v. 22.2, ANSYS Inc., Canonsburg, PA, USA), where volumetric meshes composed of tetrahedral elements were generated for each patient-specific model.

The minimum tetrahedral element size was prescribed to control the overall mesh density. A mesh independence analysis was performed by comparing velocity profiles obtained with different grid densities. Based on these analyses, meshes containing approximately 3–5 million elements, depending on the complexity of the individual geometry, were considered sufficient to accurately resolve the flow field within the patient-specific models.

To improve near-wall resolution, five prism layers were generated adjacent to the vessel walls.

### 2.4. Data Acquisition

#### 2.4.1. Flow Waveform

All included cases underwent echocardiographic examination (General Electric Vivid E95, Horten, Norway) for the diagnosis of PS, as well as for the exclusion of pulmonary artery abnormalities and the classification of patients as controls when appropriate.

For data acquisition, echocardiographic studies were retrospectively reviewed. All examinations had been performed by the Cardiology and Interventional Surgery Service of the same institution. A complete echocardiographic examination was performed in all dogs without the administration of sedative or anesthetic drugs.

**Figure 3:**
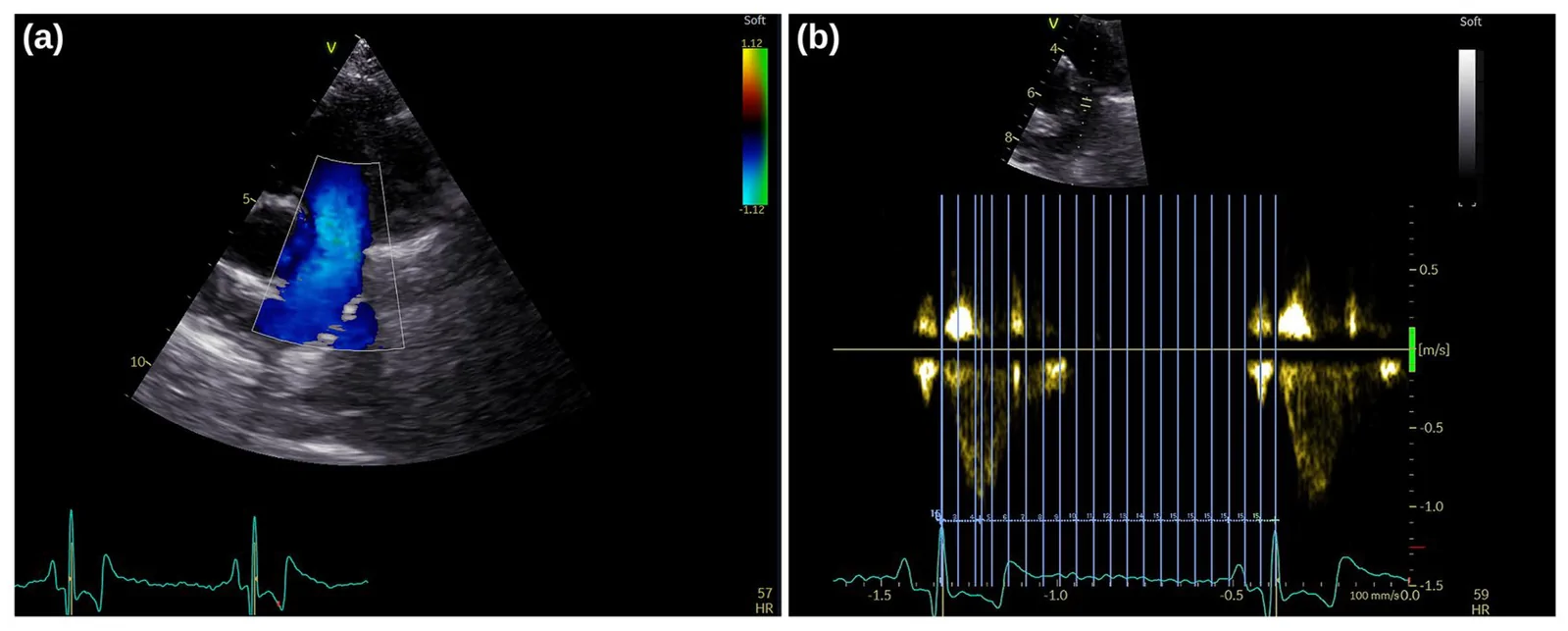
Echocardiographic assessment in case #1 from the N-PS group. (**a**) Color Doppler used to assess the direction and location of blood flow, evaluate for valvular insufficiency, and optimize the imaging plane to minimize the intercept angle. (**b**) Pulmonary artery velocity curve obtained by echocardiography, showing the division of a complete cardiac cycle into 22 time points. N-PS: non-pulmonic stenosis.

As part of the standard protocol, the anatomy of the RVOT, PV and MPA was initially assessed using two-dimensional (2D) echocardiography. Color Doppler (Figure 3a) was then performed before spectral Doppler to assess the direction and location of blood flow, evaluate for valvular insufficiency, and optimize the imaging plane to minimize the intercept angle. Pulsed-wave (PW) Doppler was subsequently used to interrogate blood flow at sequential locations within the RVOT, at the level of the PV, and immediately distal to the valve. The sample volume was moved along the outflow tract to characterize the flow profile and identify the onset and location of flow acceleration or turbulence. In dogs without PS, this assessment was used to confirm a laminar flow pattern, whereas in dogs with PS, it was used to identify the location of flow acceleration. Once aliasing and flow acceleration were identified at the level of the PV, continuous-wave (CW) Doppler was used to obtain the maximum transvalvular velocity, given the limitations of PW Doppler for assessing high-velocity blood flow. Peak velocity and pressure gradient curves across the PV were extracted from a complete cardiac cycle.

Each cardiac cycle was divided into 20 equally spaced time points, including time zero, and the peak velocity was added as an additional sampling point, resulting in a total of 22 time points per cycle. Flow velocity (m/s) and pressure gradient (mmHg) were recorded at each time point (Figure 3b). These data were used to generate patient-specific pulsatile inlet boundary conditions for the CFD simulations, including the time-varying mass flow rate and flow distribution between the LPA and RPA.

While pressure gradient is the conventional term used in echocardiography, Δp is used throughout this manuscript to describe the pressure difference across the PV, as it is more consistent with the biomechanical and CFD approach adopted in this study.

Because Doppler velocity measurements were available only at the level of the RVOT (inlet), the inlet mass flow rate (Q1) was first calculated at each time point of the cardiac cycle. The resulting inlet flow was then distributed between the LPA (Q2) and RPA (Q3) according to Murray’s law, ensuring conservation of mass (Q1 = Q2 + Q3). These outlet flow rates were subsequently used as input for the impedance-based calculation of pressure waveforms in the LPA and RPA.

Using transverse CT images and the measurement tools available in the aforementioned DICOM viewers, the inlet diameter (D_1_) at the level of the RVOT and the left (D_2_) and right (D_3_) outlet diameters were measured in meters (m). The outlet diameters were measured at the most distal point of the reconstructed LPA and RPA. The corresponding cross-sectional areas (A) were calculated as:

A = π (D_1_/2)^2^ (1)

The mass flow rate (Q, kg/s) was then calculated according to:

Q1 = ρ × A × V(2)

where ρ is the blood density (1050 kg/m^3^), A is the cross-sectional area (m^2^), and V is the blood flow velocity (m/s) measured at each time point.

The outlet flow rates were estimated according to Murray’s law [28].

For the LPA, the mass flow rate (Q2, kg/s) was calculated at each time point as:

Q2 = Q1 × (D_2_^3^/(D_2_^3^ + D_3_^3^)) (3)

For the RPA, the mass flow rate (Q3, kg/s) was calculated at each time point as:

Q3 = Q1 × (D_3_^3^/(D_2_^3^ + D_3_^3^)) (4)

#### 2.4.2. Pressure Waveforms

Pressure waveforms to be used as boundary conditions for the models were computed using the structured-tree impedance method originally developed by Olufsen (1999) [29] and Olufsen et al. (2000) [30]. By coupling each outlet to a reduced-order model of the downstream pulmonary arterial network, this approach accounts for the hemodynamic effects of the distal vasculature that are not explicitly represented in the three-dimensional reconstruction.

For each outlet of the reconstructed pulmonary artery model, the vessel radius was used as the root radius of a structured vascular tree. Starting from the terminal branches of the MPA bifurcation, two independent asymmetric binary trees were generated, representing the distal vascular territories supplied by the LPA and RPA, respectively. Because the outlet radii differed between branches, the resulting structured trees were also different.

The distal vasculature was assumed to be geometrically self-similar, with each parent vessel bifurcating into two daughter vessels according to the scaling laws proposed by Olufsen. Vessel radii were recursively calculated using Murray’s power law

(5) $$r_{p}^{j}=r_{d1}^{j}+r_{d2}^{j}$$

where ($r_{p}^{j}$) is the parent radius, ($r_{d1}^{j}$) and ($r_{d2}^{j}$) are the daughter radii, and (j = 2.76) is the scaling exponent. The recursive branching process was continued until a minimum vessel radius corresponding to the capillary level was reached.

**Figure 4:**
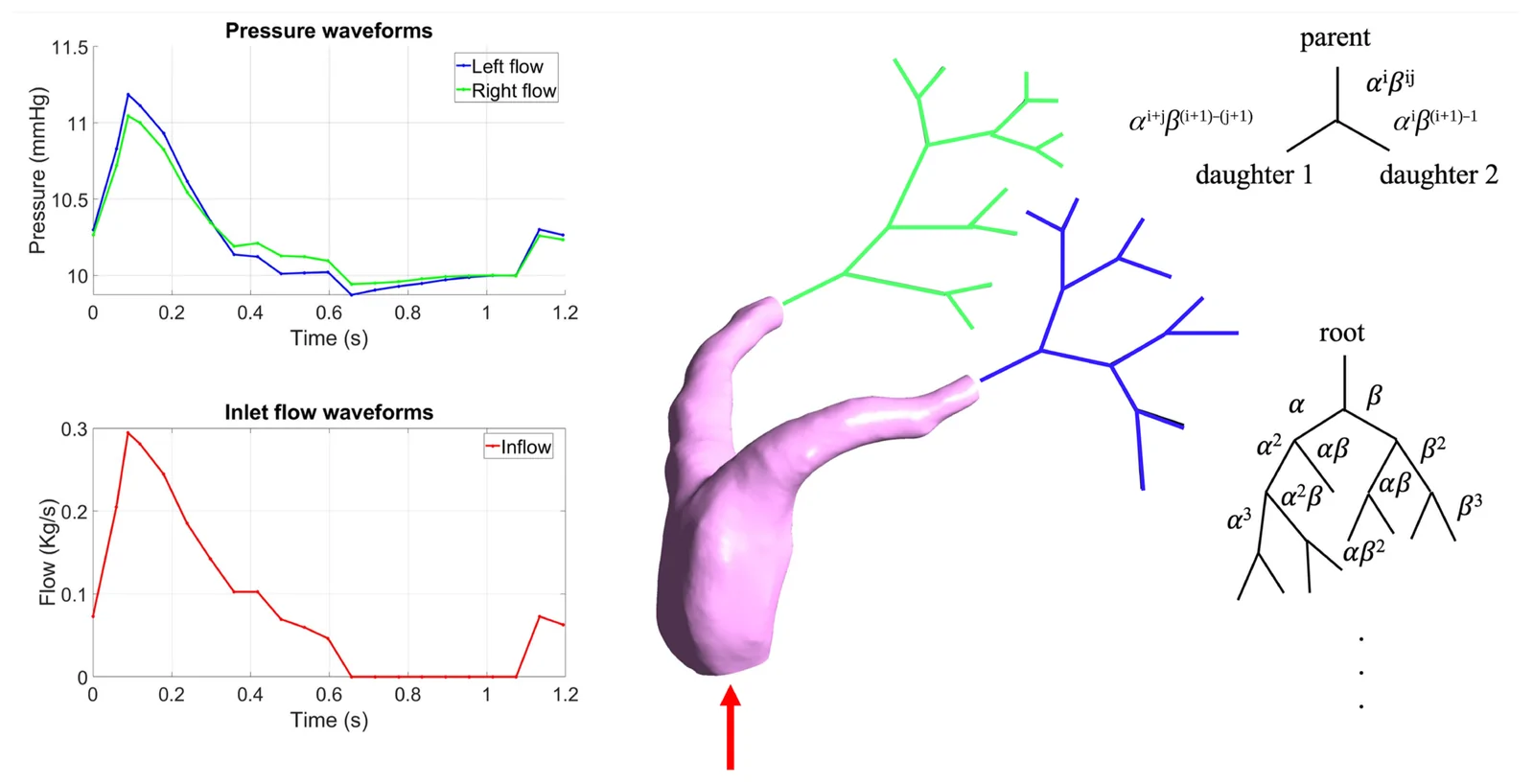
Structured tree of case #4, N-PS group (adapted from Olufsen, 1999 [29]): at each bifurcation, the radii of the daughter vessels are scaled by factors α and β. The lower left plot shows the inflow waveform. The upper left plot shows the computed impedance-based pressure waveforms. Red represents the inlet flow, green represents the pressure at the right outlet, and dark blue represents the pressure at the left outlet. N-PS: non-pulmonic stenosis.

Each parent vessel bifurcates into two daughter vessels according to scaling factors (α) and (β), which control the asymmetry of the structured tree. The radius of each vessel at generation (i) is determined recursively as

(6) $$r_{i,j}=\alpha^{i}\beta^{j-i}r_{root},0\leq j\leq i$$

where ($r_{root}$) is the radius of the root vessel. Consequently, the radii of the daughter vessels are given by

(7) $${\left(r_{0}\right)}_{d_{1}}=\alpha {\left(r_{0}\right)}_{pa},{\left(r_{0}\right)}_{d_{2}}=\beta {\left(r_{0}\right)}_{pa}$$

where ${\left(r_{0}\right)}_{pa}$ denotes the parent vessel radius. Using the radii of the outlets arising from the MPA bifurcation as root vessels, two independent asymmetric structured trees were generated to represent the distal pulmonary arterial networks supplying the left and right lungs (Figure 4).

The hemodynamic behavior of each vessel segment in the structured tree was described using the linearized one-dimensional Navier–Stokes equations. Starting from the terminal capillary vessels, where a reference pressure was prescribed, the input impedance of the downstream vascular network was recursively calculated toward the root of each tree. This procedure yielded the total frequency-dependent impedance ($Z_{tot}$) associated with every outlet of the three-dimensional model.

The total impedance of each structured tree was obtained through a recursive calculation performed from the terminal vessels toward the root of the vascular network. At the capillary level, a terminal pressure condition was prescribed, and the impedance of each vessel segment was evaluated using the linearized one-dimensional Navier–Stokes equations. For a vessel of length (L), the input impedance at its proximal end, $Z\left(0,\omega \right)$, was computed as a function of the impedance at its distal end, $Z\left(L,\omega \right)$, following the formulation proposed by Olufsen et al. (2000) [30]:

(8) $$Z\left(0,\omega \right)=\frac{i,g^{-1}\sin\left(\frac{\omega L}{c}\right)+Z\left(L,\omega \right)\cos\left(\frac{\omega L}{c}\right)}{\cos\left(\frac{\omega L}{c}\right)+igZ\left(L,\omega \right)\sin\left(\frac{\omega L}{c}\right)}$$

where (ω) is the angular frequency, (c) is the pulse wave velocity and (g) is the vessel admittance. At each bifurcation, the impedances of the daughter vessels were combined according to the parallel connection of the downstream branches, and the procedure was repeated recursively up to the root vessel. This process yielded the total frequency-dependent impedance, ($Z_{tot}$), associated with each outlet and accounting for the combined resistive, compliance and wave-propagation effects of the distal pulmonary vasculature.

Once the outlet flow waveforms, Q(t), had been obtained, the corresponding pressure waveforms were computed through convolution with the outlet impedance according to

(9) $$P\left(t\right)=\int_{t-T}^{t}Q\left(\tau \right)Z_{tot}\left(t-\tau \right)d\tau$$

where (T) is the cardiac period. The resulting pressure signals represent the hemodynamic response of the distal pulmonary vasculature and were subsequently applied as outlet boundary conditions in the three-dimensional simulations. Therefore, each outlet was subjected to a time-varying pressure boundary condition that was consistent with both the measured flow waveform and the vascular resistance and compliance of its downstream arterial tree.

### 2.5. CFD Analysis

The patient-specific computational meshes were imported into ANSYS CFX v22.2 (Ansys Inc., Canonsburg, PA, USA), where the governing fluid flow equations were solved using the finite volume method. Blood flow was modeled as unsteady throughout the cardiac cycle. The Reynolds number calculated at the model inlet exceeded 2300 in all cases; therefore, all simulations were performed under the assumption of turbulent flow. Turbulence was modeled using the Shear Stress Transport (k-ω SST) model with an initial turbulence intensity of 5%, and the flow field was obtained by solving the Reynolds-averaged Navier–Stokes (RANS) equations.

Patient-specific boundary conditions were prescribed for each simulation. At the model inlet, a transient mass flow rate waveform was imposed using the flow profile derived from Doppler velocity measurements, as described in the previous section. At the LPA and RPA outlets (first bifurcation), time-dependent pressure waveforms were prescribed to represent the hemodynamic load imposed by the distal pulmonary vasculature. These pressure boundary conditions were derived using the impedance method from the corresponding outlet flow waveforms, which had been previously distributed according to Murray’s law while satisfying mass conservation.

As a post-processing step, three-dimensional streamlines were computed to qualitatively characterize the flow structure within the pulmonary artery and its branches. The streamlines were colored according to the local velocity magnitude, allowing visualization of flow acceleration, recirculation regions, and secondary flow structures.

In addition, the main WSS-derived hemodynamic metrics were computed over one complete cardiac cycle. The time-averaged wall shear stress (TAWSS), oscillatory shear index (OSI), and relative residence time (RRT), the latter computed according to Himburg et al. (2004) [31], were calculated as follows:

(10) $$TAWSS=\left(1/T\right)\int_{0}^{T}|WSS\left(t\right)|\;dt$$

(11) $$OSI=0.5\times (1-|\int_{0}^{T}WSS\left(t\right)dt|/\int_{0}^{T}|WSS\left(t\right)|\;dt)$$

(12) $$RRT=1/\left(\left(1-2\times OSI\right)\times TAWSS\right)$$

where T is the duration of the cardiac cycle and WSS(t) is the instantaneous WSS vector.

TAWSS represents the average tangential force exerted by the flowing blood on the vessel wall throughout the cardiac cycle. It is an indicator of the overall mechanical stimulus experienced by the vascular endothelium, with low values generally associated with regions of disturbed flow.

OSI quantifies the directional changes in the WSS vector during the cardiac cycle. Values close to zero indicate a predominantly unidirectional flow, whereas values approaching 0.5 denote highly oscillatory flow with frequent reversals in the direction of WSS.

RRT combines the information provided by TAWSS and OSI to estimate the potential residence time of blood particles near the vessel wall. High RRT values identify regions where blood flow is both slow and oscillatory, conditions that may promote prolonged particle residence near the endothelium and have been associated with adverse vascular remodeling.

### 2.6. Resistance Analysis and WSS-Based Indices Evaluation

**Figure 5:**
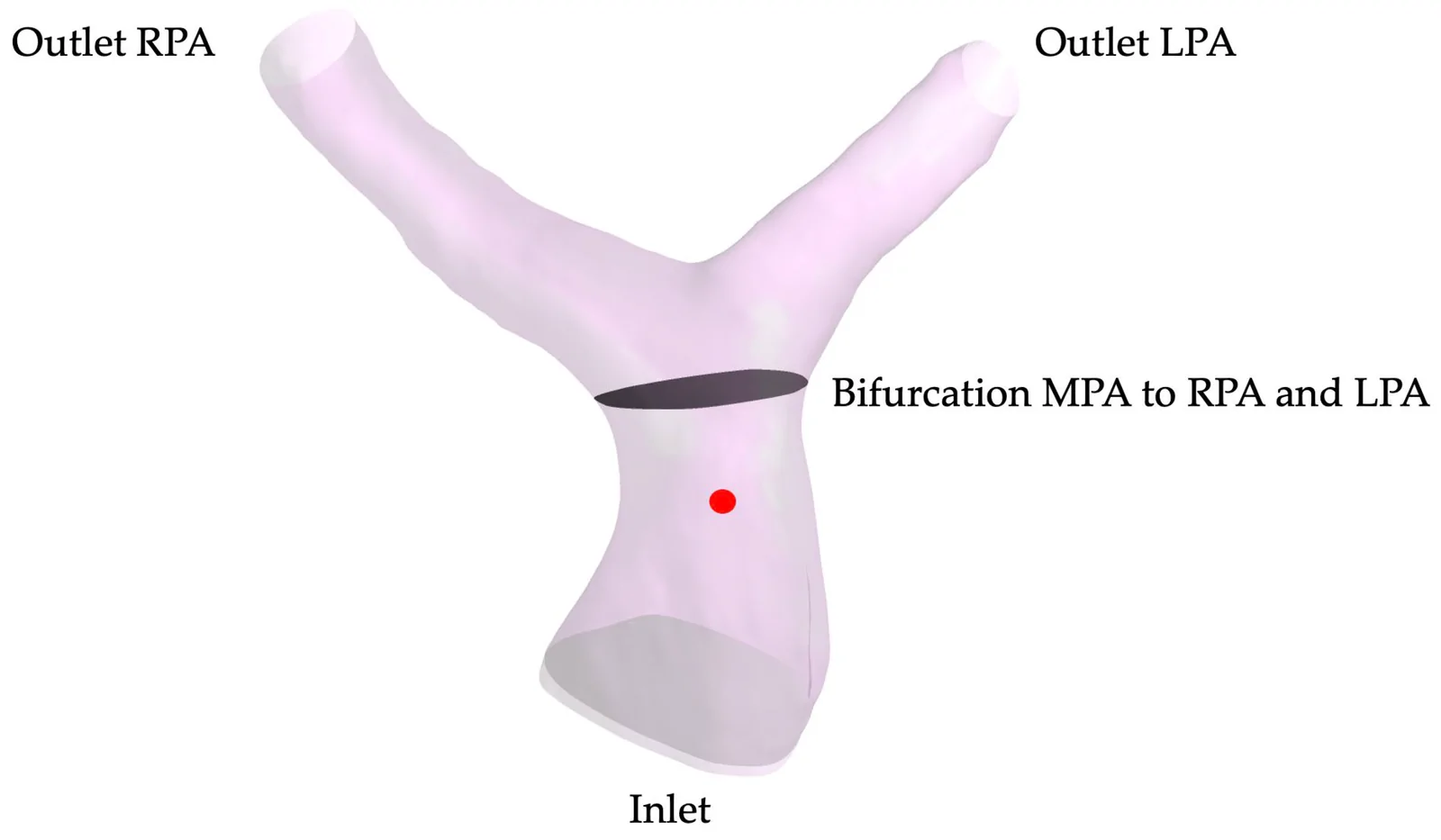
Geometric reconstruction of case #3, N-PS group, illustrating the two sectioning planes used to partition the anatomical model. The red marker indicates the predefined sampling location on the vessel wall within the reconstructed geometry, positioned 2 cm distal to the MPA bifurcation. N-PS: non-pulmonic stenosis; RPA: right pulmonary artery; LPA: left pulmonary artery; MPA: main pulmonary artery.

To quantify the local hemodynamic behavior, the computational domain was divided into predefined cross-sectional planes located at the inlet region (RVOT) and MPA immediately proximal to the bifurcation (Figure 5). Pressure values were extracted at these locations throughout the cardiac cycle.

The Δp across the MPA and PV was used to calculate the vascular resistance of each model according to:

Resistance (Pa·s/m^3^) = Δp (Pa)/flow (m^3^/s) (13)

where Δp is the pressure difference between the inlet plane (RVOT) and the plane located at the pulmonary artery bifurcation at peak systole, and the flow corresponds to the inlet flow rate at the same instant. In addition, pressure differences between the sampling planes were evaluated to characterize the pressure distribution throughout the MPA. These pressure-derived parameters were used to compare the hemodynamic burden between N-PS dogs and dogs with PS.

Maximum velocity at peak systole, mean WSS, TAWSS, OSI and RRT were also evaluated for each case. These hemodynamic parameters were extracted at a fixed point on the vessel wall, located 2 cm distal to the MPA bifurcation, allowing quantitative comparison of local flow conditions and wall mechanical loading between N-PS and PS cases.

### 2.7. Statistical Analysis

Given the descriptive nature of this study, a formal sample size calculation was not performed. The measured data were analyzed using the IBM^®^ SPSS^®^ Statistics software version 31.0.2.0 (Chicago, IL, USA). A Shapiro–Wilk test was used to test the normal distribution of age, body weight, OSI, RRT, TAWSS, mean WSS, velocity, Δp MPA (Pa and mmHg) and MPA resistance. Age and body weight were normally distributed and were summarized as mean ± standard deviation (SD) and compared between groups using an independent-samples Student’s *t*-test. The remaining continuous variables were non-normally distributed and were therefore summarized as median and interquartile range (IQR) and compared between groups using the Mann–Whitney U test. One case (case #2, PS group) was excluded from the statistical analysis because it was identified as an outlier. This dog presented the highest values of TAWSS, mean WSS, Δp across the MPA, and MPA resistance, while showing the lowest RRT value. Based on echocardiographic findings at the time of diagnosis, this case also showed the highest Δp MPA (223.330 mmHg) and peak velocity (7.490 m/s). Values of *p* < 0.05 were considered statistically significant.

## 3. Results

Ten dogs, classified as N-PS group (*n* = 5) or PS group (*n* = 5), were finally included. Within the PS group, three dogs were diagnosed with severe PS, one with moderate PS, and one with mild PS. Dogs in the N-PS group were significantly older than those in the PS group (10.20 ± 1.09 vs. 2.50 ± 4.11 years; *p* = 0.041), with ages ranging from 9 to 12 years and 4 months–9 years, respectively. Body weight did not differ significantly between groups (19.16 ± 10.13 kg in the N-PS group and 11.95 ± 6.44 kg in the PS groups; *p* = 0.258), with ranges of 6.60–28.00 kg and 5.80–21.00 kg, respectively. Signalment, clinical findings, and final diagnoses for all dogs are provided in Appendix A (Table A1).

### 3.1. Hemodynamic Characterization

**Figure 6:**
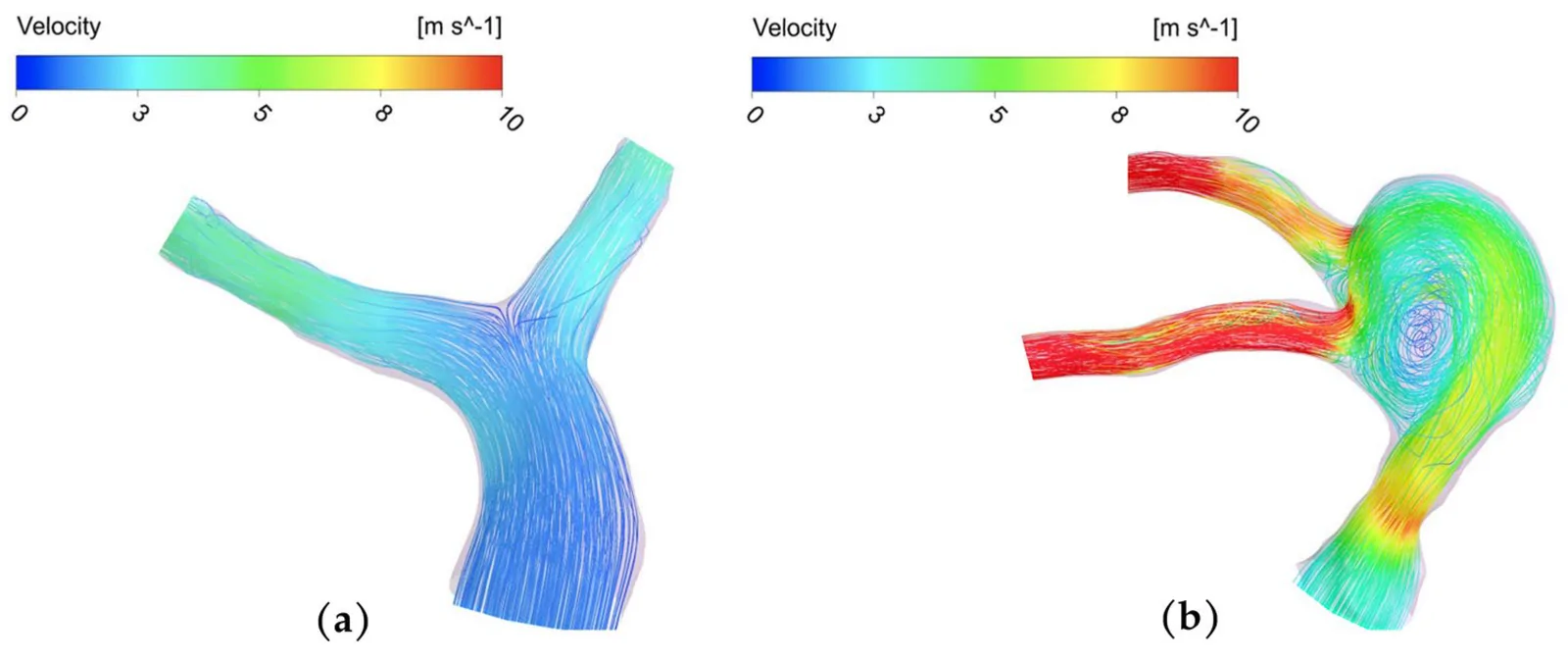
Flow streamlines represent the flow direction depicted with the intensity of the velocity (red = high velocity, dark blue = low velocity) at peak systolic velocity. N-PS case #3 (**a**) and PS (case #1) (**b**). N-PS: non-pulmonic stenosis; PS: pulmonic stenosis.

Representative flow fields obtained from CFD simulations are shown in Figure 6 for one dog without PS (Case #3, N-PS group, Figure 6a) and one dog with PS (Case #1, PS group, Figure 6b). Marked differences in flow structure were observed between the two models. The pulmonary artery in the N-PS model exhibited a relatively uniform flow pattern, with a homogeneous velocity distribution and streamlines aligned with the vessel axis throughout the MPA and its bifurcation. In contrast, the PS model showed a high-velocity jet emerging from the stenotic PV and extending into the MPA. Distal to the stenosis, the jet resulted in flow separation and localized recirculation within the post-stenotic dilatation, producing a substantially more complex flow pattern than that observed in the N-PS model.

**Figure 7:**
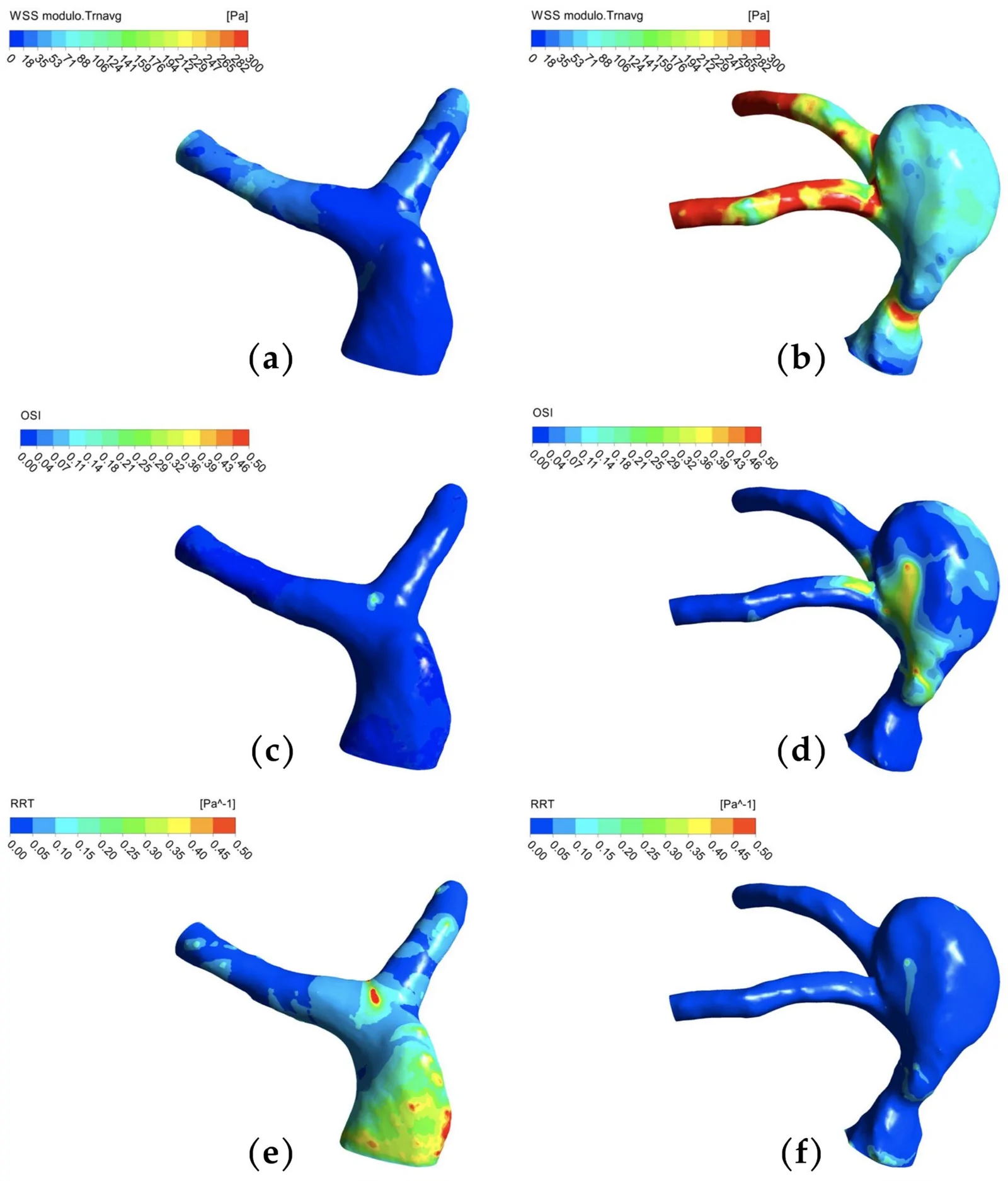
The intensity of the TAWSS (**a**,**b**), OSI (**c**,**d**) and RRT (**e**,**f**) is represented at peak systolic velocity (red = high values, dark blue = low values). Case #3, N-PS group (**a**,**c**,**e**); case #1, PS group (**b**,**d**,**f**). N-PS: non-pulmonic stenosis; PS: pulmonic stenosis; TAWSS: time-averaged wall shear stress; OSI: oscillatory shear index; RRT: relative residence time.

The increased flow velocities observed in the PS model led to marked differences in the WSS-derived indices. Regions exposed to the high-velocity jet exhibited elevated mean WSS and TAWSS values (N-PS case #3 [Figure 7a] and PS case #1 [Figure 7b]), whereas localized peaks in OSI were observed within the post-stenotic recirculation zones (N-PS case #3 [Figure 7c] and PS case #1 [Figure 7d]). Conversely, RRT values were generally lower in the PS model than in the N-PS model, despite the presence of localized disturbed flow (N-PS case #3 [Figure 7e] and PS case #1 [Figure 7f]). These representative flow characteristics were consistent with the quantitative hemodynamic differences observed between the N-PS and PS groups.

### 3.2. Quantitative Hemodynamics

**Figure 8:**
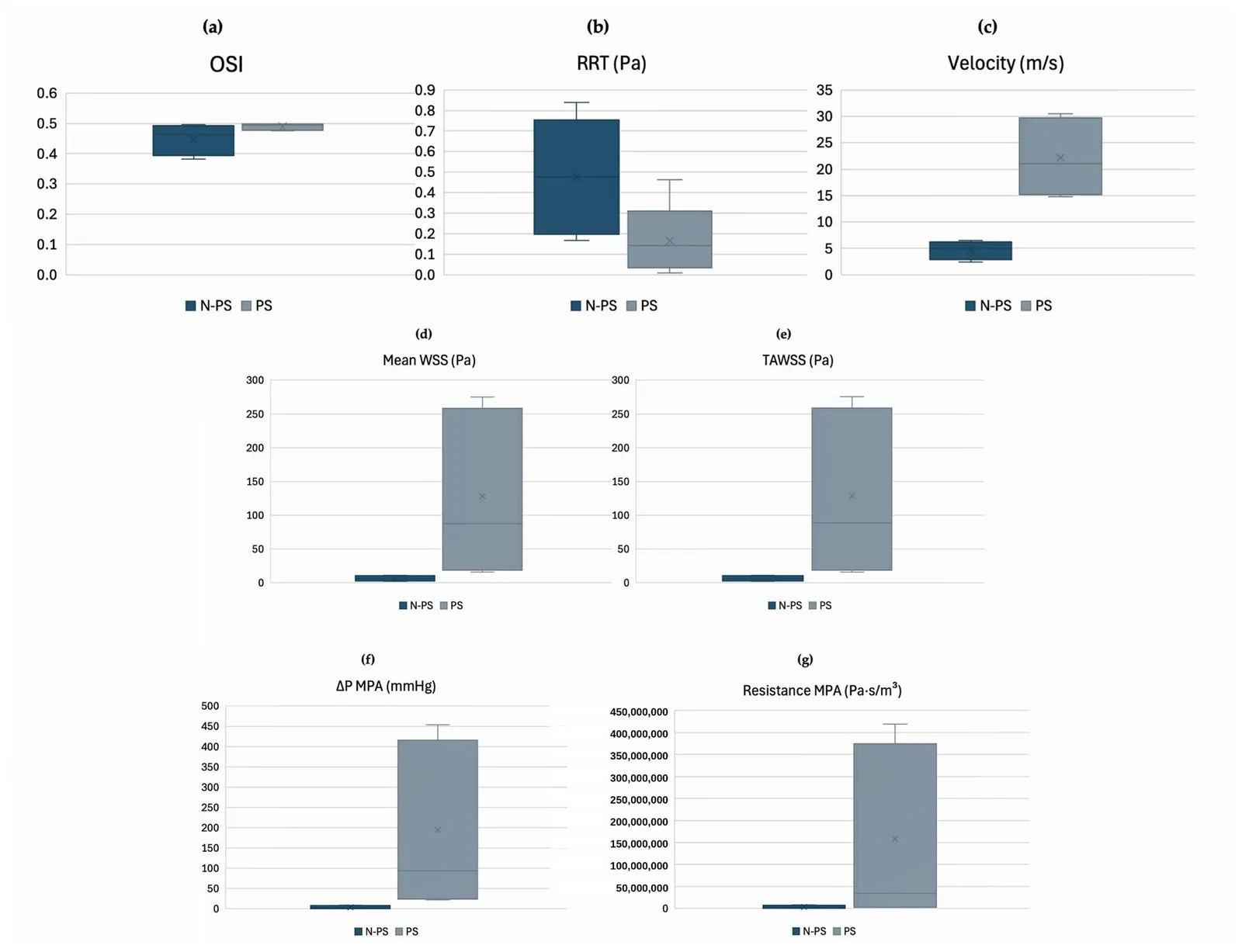
Boxplots (**a**–**g**) of results of non-parametric parameters (median and IQR). IQR: interquartile range; PS: pulmonic stenosis; N-PS: non-pulmonic stenosis; OSI: oscillatory shear index; RRT: relative residence time; TAWSS: time-averaged wall shear stress; WSS: wall shear stress; Δp: pressure drop; MPA: main pulmonary artery. The horizontal line represents the median, while the “X” represents the arithmetic mean.

All analyzed hemodynamic parameters were non-normally distributed and are presented in Figure 8. The analysis revealed statistically significant differences in hemodynamic parameters between the PS and N-PS groups, except for OSI and MPA resistance.

The median and IQR of all measured parameters for the PS and N-PS groups are represented in Appendix A (Table A2).

OSI values were similar between groups; however, the median value was higher in the PS group. All dogs had OSI values ranging from 0.38 to 0.50, approaching the upper limit of the measurement scale (Figure 8a). The normal range for OSI is 0–0.50 [32].

The median RRT was significantly higher in the N-PS group (Figure 8b).

Peak velocities (Figure 8c) were markedly higher in the PS group, with more pronounced recirculation observed in the post-stenotic dilatation. Higher velocities were observed at the stenotic region (PV) in PS cases #1, #2, and #3, which were the three cases with severe PS and higher Δp MPA values at the time of diagnosis by echocardiography.

TAWSS and mean WSS showed similar values between groups, although both were higher in the PS group (Figure 8d,e).

Pressure drop across the MPA (Figure 8f) was higher in the PS group, which also exhibited greater MPA resistance (Figure 8g). These values were higher in PS cases #1, #2, and #3, which were the cases diagnosed with severe PS.

Anatomically, PV stenosis was detected on CT in all PS cases included in this study, except for case #4, which was the only case classified as having mild stenosis on echocardiographic examination. This case exhibited the lowest TAWSS (16.0 Pa) and mean WSS (15.98 Pa) values, as well as the second-lowest MPA resistance (4,719,039.29 Pa·s/m^3^) and Δp MPA (36.02 mmHg), consistent with the echocardiographic findings (34.03 mmHg; Appendix A, Table A1).

Case #2 in the PS group differed from the other cases, exhibiting the highest values of TAWSS (484.37 Pa), mean WSS (483.08 Pa), Δp MPA (1052.91 mmHg), and MPA resistance (105,154,087.01 Pa·s/m^3^). Conversely, it showed the second-highest peak systolic velocity (28.91 m/s) and the lowest RRT value (0.0037 Pa^−1^).

## 4. Discussion

This study provides the first CFD-based characterization of pulmonary hemodynamics in dogs with and without PS. Consistent with our hypothesis, differences in hemodynamic parameters were observed between the N-PS and PS groups.

Pulmonic stenosis is a congenital disease that is typically diagnosed early in life, with previous studies reporting a median age at diagnosis of approximately 1–2 years [7,33]. Accordingly, in this study, the mean age of dogs in the PS group was lower than that of dogs in the N-PS group. The dogs without pulmonary artery abnormalities included in this study had other acquired conditions, such as bronchoesophageal fistula, chylothorax, abdominal effusion, idiopathic pericarditis, and pulmonary thromboembolism.

Valvular PS is more common in veterinary medicine than subvalvular or supravalvular forms [2], mirroring the distribution reported in human medicine [34]. Most cases included in the present study had moderate-to-severe valvular stenosis, which could be readily identified on CT and anatomically reconstructed as an irregular narrowing. In three dogs with moderate PS, the region of highest flow velocity was located at the site of stenosis, reflecting the greater degree of luminal narrowing at this point in the model.

Case #4 showed mild PS, which was not appreciable on CT images and therefore could not be represented in the anatomical reconstruction. This case exhibited the lowest TAWSS and mean WSS values within the PS group, as well as the second-lowest Δp MPA, which was consistent with the value obtained by echocardiography at the time of diagnosis. It should be noted that, in the present study, the PV was represented using a rigid or simplified reconstruction rather than a dynamic model. In CFD simulations, rigid-wall assumptions are commonly used; however, variations in vessel lumen diameter throughout the cardiac cycle are approximately 5–10% in most major normal arteries [35].

The present study employed rigid-wall CFD models based on static CT reconstructions. Although fluid–structure interaction (FSI) simulations provide a more realistic representation of cardiovascular biomechanics by accounting for vessel wall deformation and valve motion, they require substantially greater computational resources, as well as detailed information on tissue mechanical properties and boundary conditions, which are often unavailable in clinical veterinary studies [36,37]. Previous studies have shown that rigid-wall models tend to overestimate the absolute magnitude of WSS-derived metrics, whereas their spatial distribution is generally preserved [38,39]. Therefore, although the absolute values reported here should be interpreted with caution, comparisons between groups remain meaningful because all simulations were performed under the same modeling assumptions.

WSS-based descriptors, including TAWSS, OSI, and RRT, are widely used in human cardiovascular hemodynamic studies to characterize disturbed flow patterns and identify regions prone to vascular remodeling and disease development, such as atherosclerosis [40] or intracranial aneurysms [41]. These descriptors have been extensively applied to patient-specific CFD models to investigate local hemodynamic conditions [32]. For example, Sousa et al. (2014) used Doppler ultrasound-derived geometries to compare healthy and stenosed carotid bifurcations, demonstrating the ability of WSS-derived indices to identify pathological flow environments [40]. These descriptors remain valuable for characterizing the hemodynamic alterations induced by PS.

In the present study, dogs with PS exhibited higher mean WSS and TAWSS values than dogs in the N-PS group, particularly at the level of the MPA. These findings were accompanied by increased flow velocity and Δp, consistent with the echocardiographic features typically reported in dogs with PS [2,4]. Under physiological conditions, WSS is directly related to the velocity gradient at the vessel wall and therefore increases with increasing flow velocity. Consequently, the elevated TAWSS observed in the PS group most likely reflects the accelerated blood flow through the stenotic PV and the MPA. Conversely, low WSS and TAWSS values are generally associated with low flow velocities and prolonged particle residence times near the vessel wall, except in localized recirculation regions where complex flow patterns may exist [32].

All simulations in the present study assumed rigid vessel walls. Although this approach has been shown to overestimate the absolute values of WSS-related parameters compared with FSI models, the overall spatial distribution of these variables remains largely unchanged [38]. TAWSS has been reported to be approximately 21.5% lower in compliant-wall simulations of a hemodialysis arteriovenous fistula than in equivalent rigid-wall models [39]. Similar observations have been reported for the human coronary and carotid arteries, where rigid-wall models consistently overestimated TAWSS compared with compliant-wall simulations [42,43]. Therefore, although the absolute values reported here should be interpreted with caution, the comparative differences between the PS and N-PS groups are expected to remain valid because all models were generated and simulated using the same assumptions.

OSI is commonly used to identify disturbed or oscillatory flow [32]. In systemic arteries, OSI values approaching 0.5 have been associated with regions susceptible to atherosclerotic lesion development [44]. In the present study, OSI values were comparable between groups, although the PS group showed higher median values. This finding may reflect the presence of localized flow recirculation distal to the stenotic valve, particularly within the post-stenotic dilatation observed in dogs with moderate and severe PS. The coexistence of these recirculation zones and markedly increased flow velocities may have contributed to localized oscillatory WSS without substantially altering the overall OSI distribution.

RRT is a WSS-derived index that reflects the tendency of blood particles to remain in the vicinity of the vessel wall by integrating the effects of low WSS and oscillatory flow [32]. In theory, RRT ranges from zero to infinity and reaches its highest values in regions of flow stagnation and closed recirculation, where OSI approaches 0.5 [32]. Previous studies have suggested that RRT values above 8 Pa^−1^ should be considered elevated [45]. In the present study, however, none of the dogs exhibited RRT values exceeding 0.8 Pa^−1^. Interestingly, median RRT values were lower in the PS group than in the N-PS group. Although this observation differs from findings reported in systemic arteries, where elevated RRT is typically associated with pathological recirculation regions, it may reflect the distinct hemodynamic environment produced by PS. Specifically, the marked increase in TAWSS generated by the high-velocity jet downstream of the stenotic PV likely outweighed the modest increase in OSI, resulting in lower overall RRT values despite the presence of localized post-stenotic recirculation. Therefore, although recirculation zones were observed distal to the stenosis, the accelerated bulk flow may have reduced the overall tendency of blood particles to remain near the vessel wall.

The Δp between the RVOT and the bifurcation of the RPA and LPA was significantly higher in dogs with PS than in the control group, consistent with the findings commonly reported by echocardiography in these patients at the time of diagnosis [2]. In clinical practice, elevated peak flow velocities are converted into an estimated Δp using the simplified Bernoulli equation [37]. Echocardiographic assessment is typically performed using Doppler interrogation across the PV. Accordingly, the CFD models used in the present study enabled the reconstruction and quantification of the Δp across the stenotic point, encompassing the pre-valvular and post-valvular regions. MPA resistance was calculated using these data and was increased in dogs with PS compared with controls. The highest values of these two hemodynamic parameters were reported in the three cases diagnosed with severe PS, meaning Δp > 80 mmHg [2]. In both human and veterinary medicine, the available literature is limited to studies evaluating distal vascular resistance in the context of systemic hypertension or pulmonary vascular resistance. Consequently, direct comparison with the present results is not feasible [46,47].

Within the PS group, one case exhibited hemodynamic parameters that markedly deviated from those observed in the remaining cases and was therefore excluded from the analysis as an outlier. This dog had severe mixed valvular PS (types A and B) associated with tricuspid dysplasia. The markedly elevated hemodynamic parameters observed in this case may have affected the boundary conditions applied, particularly in conjunction with the rigid anatomical model used in the present study. Given the pilot nature of this study and the limited number of comparable cases, these findings cannot be reliably contextualized against the existing literature.

This study has several limitations that should be considered when interpreting the results. First, the relatively small sample size and retrospective clinical study design may limit the generalizability of the findings. In addition, the exclusion of cases with incomplete echocardiographic data may have introduced selection bias. Variability in CT acquisition parameters, particularly slice thickness, together with mild respiratory and cardiac motion artifacts, may also have influenced the accuracy of the anatomical reconstructions. It should be acknowledged that the drugs administered to the patients may have influenced cardiac output or other hemodynamic parameters to some extent. Nonetheless, given that CT imaging was employed solely for anatomical assessment and vessel reconstruction, this potential influence is considered negligible in terms of the geometry obtained.

An additional limitation is the clinical heterogeneity of the N-PS group. Some dogs had concurrent cardiac conditions; however, echocardiographic examination ruled out PV abnormalities, cardiac remodeling, evidence of pulmonary hypertension, and systolic or diastolic dysfunction. Nevertheless, given the retrospective nature of the study, inclusion was limited to dogs for which both echocardiography and thoracic CT were available as part of the diagnostic work-up. Consequently, the N-PS group may have been subject to selection bias, as these dogs were not necessarily representative of a healthy population and had initially undergone diagnostic evaluation because of an underlying clinical condition.

Furthermore, the CFD simulations were based on static CT images acquired without ECG-gating. Consequently, the reconstructed geometries represent a single anatomical configuration and do not account for the dynamic motion of the PV or vessel walls throughout the cardiac cycle. The assumption of rigid walls neglects FSI effects, which have been shown to influence the absolute magnitude of hemodynamic parameters such as WSS and TAWSS, although their impact on the overall spatial distribution is generally less pronounced.

Finally, invasive catheter-derived pressure measurements, considered the reference standard for hemodynamic assessment in human medicine, were not available for all dogs included in this study. Therefore, Δp values were derived exclusively from echocardiographic examinations. Although invasive pressure measurements were available in a subset of dogs undergoing PBV, these data were not consistently recorded and were excluded to ensure methodological consistency across the study population.

Future studies involving larger prospective cohorts, ECG-gated dynamic imaging, FSI simulations, and systematic acquisition of invasive hemodynamic measurements would help validate and extend the findings reported here.

## 5. Conclusions

This study demonstrates the feasibility of patient-specific CFD for characterizing pulmonary artery hemodynamics in dogs with and without PS. Dogs with PS exhibited marked alterations in flow patterns, including increased flow velocity, elevated Δp values, and the presence of post-stenotic flow disturbances compared with controls. These hemodynamic alterations, characteristic of blood flow behavior in valvular stenosis and post-stenotic changes, were further evidenced by WSS-related parameters, specifically elevated mean WSS and reduced RRT values in the PS group.

Overall, the results suggest that PS leads to significant modifications in pulmonary artery blood flow dynamics that are not fully captured by conventional imaging techniques alone. Patient-specific CFD may therefore provide complementary information for the evaluation of disease severity and improve the understanding of the biomechanical consequences of pulmonary outflow tract obstruction in dogs. These insights could serve as a basis for further investigation into the potential role of CFD in surgical planning, the development of novel therapeutic approaches, and prognostic assessment based on the specific behavior and characteristics of the evaluated flow. Further studies including larger populations are warranted to validate these findings and explore their clinical application and relevance.

## Data Availability

All data are included in this article and Supplementary Materials.

## Supplementary Materials

The following supporting information can be downloaded at https://www.mdpi.com/article/10.3390/ani16182842/s1, Figure S1: Flow maps of all cases using streamlines and colored with the velocity intensity (m/s). Airflow streamlines represent the flow direction depicted with the intensity of the velocity (red = high velocity, dark blue = low velocity) at peak systolic velocity. N-PS: non-pulmonic stenosis; PS: pulmonic stenosis; Figure S2: TAWSS maps of all cases. The intensity of the pressure is represented at peak systolic velocity (red = high pressure, dark blue = low pressure). TAWSS: time average wall shear stress; N-PS: non-pulmonic stenosis; PS: pulmonic stenosis; Figure S3: Mean WSS maps of all cases. The intensity of the pressure is represented at peak systolic velocity (red = high pressure, dark blue = low pressure). WSS: wall shear stress; N-PS: non- pulmonic stenosis; PS: pulmonic stenosis; Figure S4: RRT maps of all cases. The intensity of the pressure is represented at peak systolic velocity (red = high pressure, dark blue = low pressure). RRT: relative residence time; N-PS: non-pulmonic stenosis; PS: pulmonic stenosis.; Figure S5: OSI maps of all cases. The intensity of the value is represented at peak systolic velocity (red = high pressure, dark blue = low pressure). OSI: oscillatory shear index. N-PS: non-pulmonic stenosis; PS: pulmonic stenosis.

## Abbreviations

The following abbreviations are used in this manuscript:

PS	Pulmonic stenosis
MPA	Main pulmonary artery
PV	Pulmonary valve
PBV	Pulmonary balloon valvuloplasty
ECG	Electrocardiographic
CTA	Computed tomographic angiography
MRI	Magnetic resonance imaging
CFD	Computational fluid dynamics
WSS	Wall shear stress
Δp	Pressure drop
CT	Computed tomography
N-PS	Non-pulmonic stenosis
MAP	Main arterial pressure
DICOM	Digital Imaging and Communications in Medicine
RVOT	Right ventricular outflow tract
LPA	Left pulmonary artery
RPA	Right pulmonary artery
STL	stereolithography
SST	Shear Stress Transport
RANS	Reynolds-Averaged Navier–Stokes
TAWSS	Time-averaged wall shear stress
OSI	Oscillatory shear index
RRT	Relative residence time
SD	Standard deviation
IQR	Interquartile range
FSI	Fluid–structure interaction

## Appendix A

**Table A1:** Information on breed, body weight, gender, age, clinical signs and final diagnosis for each dog included.

PS Group	Breed	Body Weight (kg)	Gender	Age	Clinical Signs	Final Diagnosis
**Case #1**	Brittany	5.8	F	4 mo	No clinical signs Cardiac murmur	Severe valvular PS type A (pressure of 170 mmHg)
**Case #2**	Crossbreed	19	M-N	9 mo	PS evaluation	Severe valvular PS type A and B (pressure of 223 mmHg) Tricuspid dysplasia
**Case #3**	Spanish Water Dog	10	F	5 mo	Respiratory distress	Severe valvular PS type A (pressure of 115 mmHg) Cor triatriatum dexter and interatrial septal defect
**Case #4**	French Bulldog	11	M-N	9 yr	3rd-degree block	Mild valvular PS type A (pressure of 34 mmHg) Pulmonary hypertension Myxomatous mitral valve disease
**Case #5**	Golden Retriever	21	M	2 yr	Pre-syncopal collapse	Moderate to severe valvular PS type A (pressure of 67 mmHg) Tricuspid dysplasia Severe right ventricular remodeling and dysfunction
**N-PS group**						
**Case #1**	Crossbreed	6.6	F-N	10 yr	Chronic cough	Bronchoesophageal fistula
**Case #2**	American Pit Bull	24.9	F	12 yr	Ascites	Modified transudate. No cardiac cause was identified
**Case #3**	WHWT	9.8	F	10 yr	Exercise intolerance	Chylothorax and thromboembolism of the external jugular veins
**Case #4**	Crossbreed	26.5	F-N	10 yr	Mild pericardial effusion	Idiopathic pericarditis. No structural cardiac abnormalities were seen
**Case #5**	Akita Inu	28	F-N	9 yr	Thrombocytopenia Chronic enteropathy	Pulmonary thromboembolism

Notes. PS: pulmonic stenosis; N-PS: non-pulmonic stenosis; F: female; F-N: female neutered; M: male; M-N: male neutered; mo: months; yr: years; WHWT: West Highland White Terrier; # represents the case number.

**Table A2:** Results of the variables represented by median (IQR) and the statistical significance represented by *p*-value.

Variable	N-PS Group	PS Group	*p*-Value
OSI	0.463	0.495	0.190
	(0.407–0.490)	(0.480–0.497)	
RRT (Pa^−1^)	0.230	0.057	0.032
	(0.167–0.669)	(0.010–0.143)	
TAWSS (Pa)	5.760	88.438	0.016
	(2.459–10.084)	(20.981–241.998)	
Mean WSS (Pa)	5.759	87.816	0.016
	(2.449–10.057)	(20.921–241.323)	
Velocity (m/s)	4.998	20.995	0.016
	(3.515–5.922)	(15.520–28.951)	
Δp MPA (Pa)	271.990	12,523.000	0.016
	(67.765–878.000)	(3375.500–50,385.000)	
Δp MPA (mmHg)	2.040	93.931	0.016
	(0.509–6.586)	(25.318–377.920)	
Resistance MPA (Pa·s/m^3^)	2,143,679.519	32,518,820.16	0.190
	(228,841.389–6,331,035.006)	(2,844,385.569–330,188,411.586)	

Notes. IQR: interquartile range; PS: pulmonic stenosis; N-PS: non-pulmonic stenosis; OSI: oscillatory shear index; RRT: relative residence time; TAWSS: time-averaged wall shear stress; WSS: wall shear stress; Δp: pressure drop; MPA: main pulmonary artery.

## References

[1] Schrope D.P. (2015). Prevalence of Congenital Heart Disease in 76,301 Mixed-Breed Dogs and 57,025 Mixed-Breed Cats. J. Vet. Cardiol..

[2] Bussadori C., Amberger C., Le Bobinnec G., Lombard C.W. (2000). Guidelines for the echocardiographic studies of suspected subaortic and pulmonic stenosis. J. Vet. Cardiol..

[3] Bussadori C., Bussadori C. (2022). Congenital Diseases of the Right Heart. Textbook of Cardiovascular Medicine in Dogs and Cats.

[4] Nishimura S., Visser L.C., Bélanger C., Oldach M.S., Gunther-Harrington C.T., Stern J.A. (2018). Echocardiographic Evaluation of Velocity Ratio, Velocity Time Integral Ratio, and Pulmonary Valve Area in Dogs with Pulmonary Valve Stenosis. J. Vet. Intern. Med..

[5] Brown D.J., Smith F.W.K. (2002). Stenosis Hemodynamics: From Physical Principles to Clinical Indices. J. Vet. Intern. Med..

[6] Gunther-Harrington C.T., Phillips K.L., Visser L.C., Fousse S.L., Stern J.A. (2019). Non-Electrocardiographic-Gated Computed Tomographic Angiography Can Be Used to Diagnose Coronary Artery Anomalies in Bulldogs with Pulmonary Valve Stenosis. Vet. Radiol. Ultrasound.

[7] Bussadori C., Demadron E., Santilli R.A., Borgarelli M. (2001). Balloon Valvuloplasty in 30 Dogs with Pulmonic Stenosis: Effect of Valve Morphology and Annular Size on Initial and 1-Year Outcome. J. Vet. Intern. Med..

[8] Locatelli C., Spalla I., Domenech O., Sala E., Brambilla P.G., Bussadori C. (2013). Pulmonic Stenosis in Dogs: Survival and Risk Factors in a Retrospective Cohort of Patients. J. Small Anim. Pract..

[9] Drees R., Frydrychowicz A., Reeder S.B., Pinkerton M.E., Johnson R. (2011). 64-Multidetector Computed Tomographic Angiography of the Canine Coronary Arteries. Vet. Radiol. Ultrasound.

[10] Laborda-Vidal P., Pedro B., Baker M., Gelzer A.R., Dukes-McEwan J., Maddox T.W. (2016). Use of ECG-Gated Computed Tomography, Echocardiography and Selective Angiography in Five Dogs with Pulmonic Stenosis and One Dog with Pulmonic Stenosis and Aberrant Coronary Arteries. J. Vet. Cardiol..

[11] Drees R., François C.J., Saunders J.H. (2014). Invited Review-Computed Tomographic Angiography (CTA) of the Thoracic Cardiovascular System in Companion Animals. Vet. Radiol. Ultrasound.

[12] Laborda-Vidal P., Maddox T.W., Navarro-Cubas X., Dukes-McEwan J., McConnell J.F. (2015). Comparison between Echocardiographic and Non-ECG-Gated CT Measurements in Dogs. Vet. Rec..

[13] Gerrah R., Haller S.J., George I. (2017). Mechanical Concepts Applied in Congenital Heart Disease and Cardiac Surgery. Ann. Thorac. Surg..

[14] Hsia T.Y., Figliola R., Bove E., Dorfman A., Taylor A., Giardini A., Khambadkone S., Schievano S., Baker G.H., Hlavacek A. (2016). Multiscale Modelling of Single-Ventricle Hearts for Clinical Decision Support: A Leducq Transatlantic Network of Excellence. Eur. J. Cardiothorac. Surg..

[15] Gerrah R., Haller S.J. (2020). Computational Fluid Dynamics: A Primer for Congenital Heart Disease Clinicians. Asian Cardiovasc. Thorac. Ann..

[16] Del Álamo J.C., Marsden A.L., Lasheras J.C., Lasheras J.C. (2009). Recent Advances in the Application of Computational Mechanics to the Diagnosis and Treatment of Cardiovascular Disease. Rev. Esp. Cardiol..

[17] Basri E.I., Riazuddin V.N., Azriff A., Zuber M., Azriff Basri A., Shahwir S.F., Ahmad K.A. (2016). Computational Fluid Dynamics Study in Biomedical Applications: A Review. Int. J. Fluids Heat Transf..

[18] Devlin C., Tomov M.L., Chen H., Nama S., Ali S., Neelakantan S., Avazmohammadi R., Dasi L.P., Bauser-Heaton H.D., Serpooshan V. (2024). Patient-Specific 3D in Vitro Modeling and Fluid Dynamic Analysis of Primary Pulmonary Vein Stenosis. Front. Cardiovasc. Med..

[19] Conijn M., Krings G.J. (2021). Computational Analysis of the Pulmonary Arteries in Congenital Heart Disease: A Review of the Methods and Results. Comput. Math. Methods Med..

[20] Hostnik E.T., Scansen B.A., Zielinski R., Ghadiali S.N. (2017). Quantification of Nasal Airflow Resistance in English Bulldogs Using Computed Tomography and Computational Fluid Dynamics. Vet. Radiol. Ultrasound.

[21] Khoa N.D., Phuong N.L., Tani K., Inthavong K., Ito K. (2023). In-Silico Decongested Trial Effects on the Impaired Breathing Function of a Bulldog Suffering from Severe Brachycephalic Obstructive Airway Syndrome. Comput. Methods Programs Biomed..

[22] Khoa N.D., Phuong N.L., Tani K., Inthavong K., Ito K. (2021). Computational Fluid Dynamics Comparison of Impaired Breathing Function in French Bulldogs with Nostril Stenosis and an Examination of the Efficacy of Rhinoplasty. Comput. Biol. Med..

[23] Fernández-Parra R., Pey P., Zilberstein L., Malvè M. (2019). Use of Computational Fluid Dynamics to Compare Upper Airway Pressures and Airflow Resistance in Brachycephalic, Mesocephalic, and Dolichocephalic Dogs. Vet. J..

[24] Fernández-Parra R., Pey P., Reinero C., Malvè M. (2023). Salbutamol Transport and Deposition in Healthy Cat Airways under Different Breathing Conditions and Particle Sizes. Front. Vet. Sci..

[25] Fernández-Parra R., Pey P., Reinero C., Malvè M. (2021). Salbutamol Transport and Deposition in the Upper and Lower Airway with Different Devices in Cats: A Computational Fluid Dynamics Approach. Animals.

[26] Zamora-Perarnau C., Malvè M., Fernández-Parra R. (2026). Numerical Modelling of Airflow and Aerosol Particle Delivery in Cats with Bronchial and Non-Bronchial Disease. J. Biomech..

[27] Zamora-Perarnau C., Malvè M., Fernández-Parra R. (2023). Computational Fluid Dynamics Comparison of the Upper Airway Velocity, Pressure, and Resistance in Cats Using an Endotracheal Tube or a Supraglottic Airway Device. Front. Vet. Sci..

[28] Murray C.D. (1926). The Physiological Principle of Minimum Work. Proc. Natl. Acad. Sci. USA.

[29] Olufsen M.S. (1999). Structured Tree Outflow Condition for Blood Flow in Larger Systemic Arteries. Am. J. Physiol..

[30] Olufsen M.S., Peskin C.S., Kim W.Y., Pedersen E.M., Nadim A., Larsen J. (2000). Numerical Simulation and Experimental Validation of Blood Flow in Arteries with Structured-Tree Outflow Conditions. Ann. Biomed. Eng..

[31] Himburg H.A., Grzybowski D.M., Hazel A.L., Lamack J.A., Li X.M., Friedman M.H. (2004). Spatial comparison between wall shear stress measures and porcine arterial endothelial permeability. Am. J. Physiol. Heart Circ. Physiol..

[32] Pinto S.I.S., Campos J.B.L.M. (2016). Numerical Study of Wall Shear Stress-Based Descriptors in the Human Left Coronary Artery. Comput. Methods Biomech. Biomed. Eng..

[33] Bini M., Vezzosi T., Del Palacio M.J.F., Talavera J., Patata V., Marchesotti F., Domenech O. (2022). Clinical and Electrocardiographic Findings for Predicting the Severity of Pulmonary Valve Stenosis in Dogs. Vet. Sci..

[34] Fathallah M., Krasuski R.A. (2017). Pulmonic Valve Disease: Review of Pathology and Current Treatment Options. Curr. Cardiol. Rep..

[35] Taylor C.A., Hughes T.J.R., Zarins C.K. (1998). Finite Element Modeling of Three-Dimensional Pulsatile Flow in the Abdominal Aorta: Relevance to Atherosclerosis. Ann. Biomed. Eng..

[36] Taylor C.A., Figueroa C.A. (2009). Patient-Specific Modeling of Cardiovascular Mechanics. Annu. Rev. Biomed. Eng..

[37] Morris P.D., Narracott A., Von Tengg-Kobligk H., Soto D.A.S., Hsiao S., Lungu A., Evans P., Bressloff N.W., Lawford P.V., Rodney Hose D. (2016). Computational Fluid Dynamics Modelling in Cardiovascular Medicine. Heart.

[38] Kim Y.H., Kim J.E., Ito Y., Shih A.M., Brott B., Anayiotos A. (2008). Hemodynamic Analysis of a Compliant Femoral Artery Bifurcation Model Using a Fluid Structure Interaction Framework. Ann. Biomed. Eng..

[39] McGah P.M., Leotta D.F., Beach K.W., Aliseda A. (2014). Effects of Wall Distensibility in Hemodynamic Simulations of an Arteriovenous Fistula. Biomech. Model. Mechanobiol..

[40] Sousa L.C., Castro C.F., António C.C., Santos A., Santos R., Castro P., Azevedo E., Tavares J.M.R.S. (2014). Haemodynamic Conditions of Patient-Specific Carotid Bifurcation Based on Ultrasound Imaging. Comput. Methods Biomech. Biomed. Eng. Imaging.

[41] Loly V.T.R., Cintra A., Ramirez-Velandia F., Ogilvy C.S., Mensah E.O., de Sá Brasil Lima J., Nucci M.P., Baccin C.E., Gamarra L.F. (2025). Computational Fluid Dynamics Approaches for Analyzing Rupture and Growth of Intracranial Aneurysms: A Systematic Review. Biomedicines.

[42] Malvè M., García A., Ohayon J., Martínez M.A. (2012). Unsteady Blood Flow and Mass Transfer of a Human Left Coronary Artery Bifurcation: FSI vs. CFD. Int. Commun. Heat Mass Transf..

[43] Malvè M., Chandra S., García A., Mena A., Martínez M.A., Finol E.A., Doblaré M. (2014). Impedance-Based Outflow Boundary Conditions for Human Carotid Haemodynamics. Comput. Methods Biomech. Biomed. Eng..

[44] He X., Ku D.N. (1996). Pulsatile Flow in the Human Left Coronary Artery Bifurcation: Average Conditions. J. Biomech. Eng..

[45] Lee S.W., Antiga L., Steinman D.A. (2009). Correlations among Indicators of Disturbed Flow at the Normal Carotid Bifurcation. J. Biomech. Eng..

[46] Chemla D., Lau E.M.T., Papelier Y., Attal P., Hervé P. (2015). Pulmonary vascular resistance and compliance relationship in pulmonary hypertension. Eur. Respir. J..

[47] Kwan W.C., Shavelle D.M., Laughrun D.R. (2019). Pulmonary Vascular Resistance Index: Getting the Units Right and Why It Matters. Clin. Cardiol..

