# Supplementary material for: Patient-Specific Computational Fluid Dynamics of Pulmonary Arterial Hemodynamics in Dogs with and Without Pulmonic Stenosis
Source: Animals (Basel). 2026 Sep 9;16(18):2842. doi: 10.3390/ani16182842 (PMC13603379; doi:10.3390/ani16182842)
Supplement: Supplementary file 1 [file animals-16-02842-s001.zip › animals-4461323-supplementary.pdf]

Supplementary material

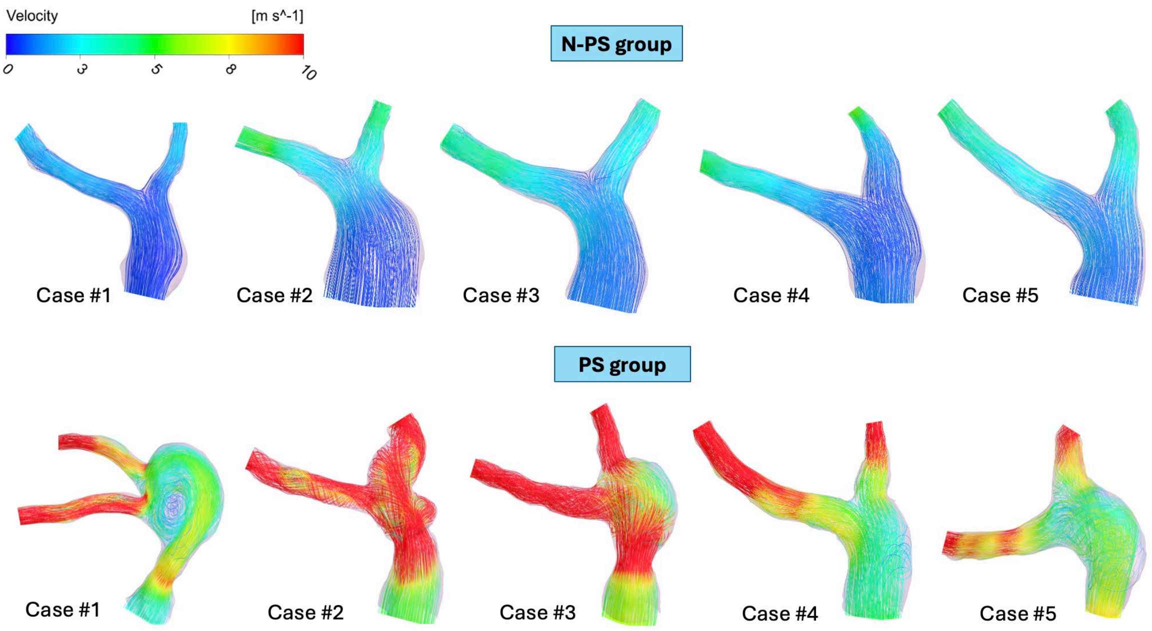

Figure S1. Flow maps of all cases using streamlines and colored with the velocity intensity (m/s). Airflow streamlines represent the flow direction depicted with the intensity of the velocity (red = high velocity, dark blue = low velocity) at peak systolic velocity. N-PS: non pulmonic stenosis; PS: pulmonic stenosis.

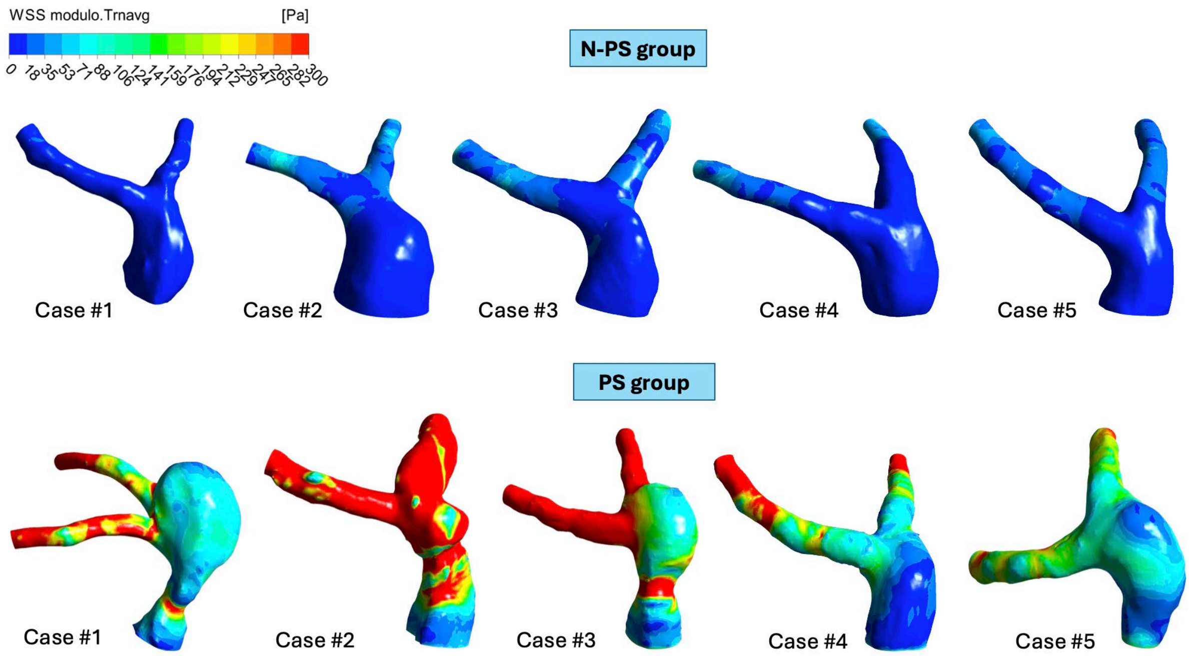

Figure S2. TAWSS maps of all cases. The intensity of the pressure is represented at peak systolic velocity (red = high pressure, dark blue = low pressure). TAWSS: time average wall shear stress; N-PS: non pulmonic stenosis; PS: pulmonic stenosis.

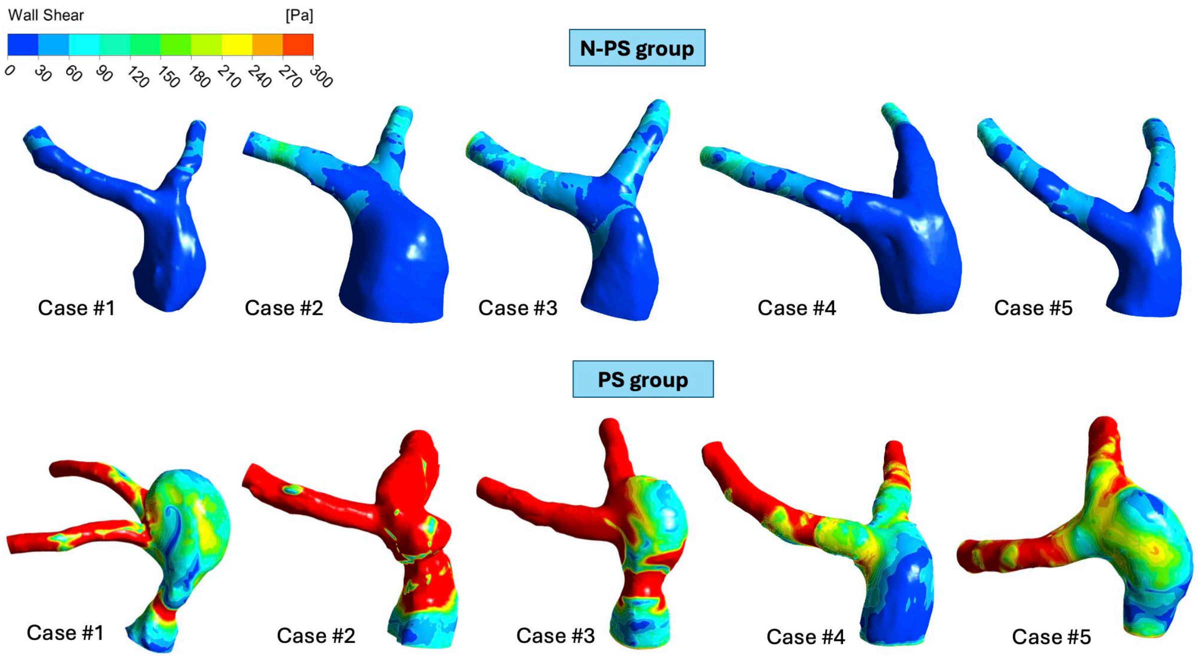

Figure S3. Mean WSS maps of all cases. The intensity of the pressure is represented at peak systolic velocity (red = high pressure, dark blue = low pressure). WSS: wall shear stress; N-PS: non pulmonic stenosis; PS: pulmonic stenosis.

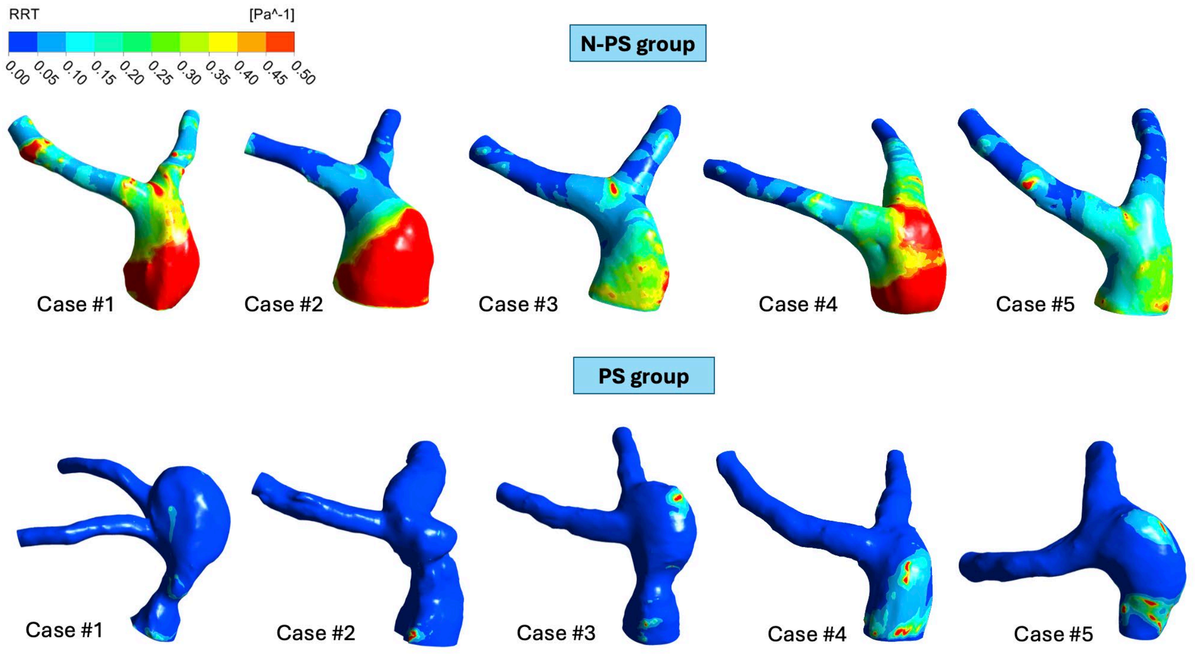

Figure S4. RRT maps of all cases. The intensity of the pressure is represented at peak systolic velocity (red = high pressure, dark blue = low pressure). RRT: relative residence time; N-PS: non pulmonic stenosis; PS: pulmonic stenosis.

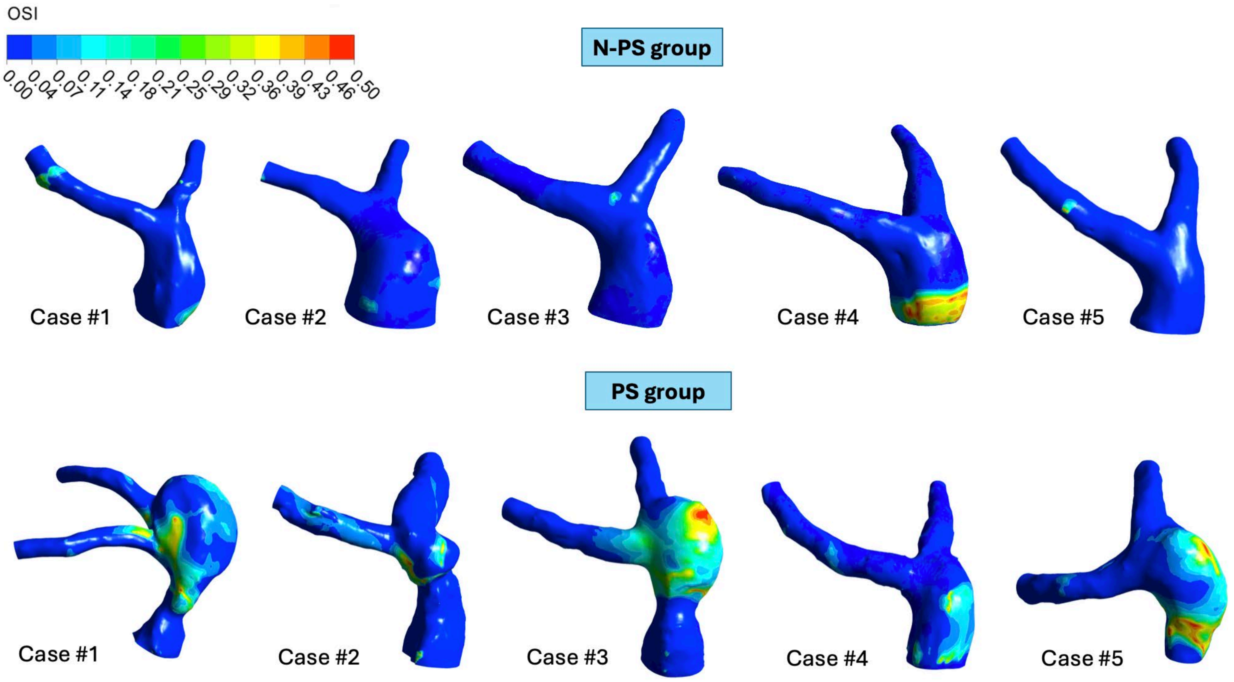

Figure S5. OSI maps of all cases. The intensity of the value is represented at peak systolic velocity (red = high pressure, dark blue = low pressure). OSI: oscillatory shear index. N-PS: non pulmonic stenosis; PS: pulmonic stenosis.
